# Genomic and transcriptomic profiling reveal molecular characteristics of parathyroid carcinoma

**DOI:** 10.1038/s12276-023-00968-4

**Published:** 2023-05-01

**Authors:** Se-Young Jo, Namki Hong, Seunghyun Lee, Jong Ju Jeong, Jeongsoo Won, Jiho Park, Gi Jeong Kim, Sang Kyum Kim, Sangwoo Kim, Yumie Rhee

**Affiliations:** 1 Department of Biomedical Systems Informatics, Yonsei University College of Medicine, Seoul, Korea; 2 Brain Korea 21 PLUS Project for Medical Science, Yonsei University College of Medicine, Seoul, Korea; 3 Department of Internal Medicine, Severance Hospital, Endocrine Research Institute, Yonsei University College of Medicine, Seoul, South Korea; 4 Department of Internal Medicine, Wonju Severance Christian Hospital, Yonsei University Wonju College of Medicine, Wonju, Korea; 5 Department of Surgery, Severance Hospital, Yonsei University College of Medicine, Seoul, Korea; 6 Department of Pathology, Yonsei University College of Medicine, Seoul, Korea; 7 Postech Biotech Center, Pohang University of Science and Technology (POSTECH), Pohang, Korea

**Keywords:** Cancer genomics, Genetics research, Classification and taxonomy

## Abstract

Genomic and transcriptomic profiling has enhanced the diagnostic and treatment options for many cancers. However, the molecular characteristics of parathyroid cancer remain largely unexplored, thereby limiting the development of new therapeutic interventions. Herein, we conducted genomic and transcriptomic sequencing of 50 parathyroid tissues (12 carcinomas, 28 adenomas, and 10 normal tissues) to investigate the intrinsic and comparative molecular features of parathyroid carcinoma. We confirmed multiple two-hit mutation patterns in cell division cycle 73 (*CDC73*) that converged to biallelic inactivation, calling into question the presence of a second hit in other genes. In addition, allele-specific repression of *CDC73* in copies with germline-truncating variants suggested selective pressure prior to tumorigenesis. Transcriptomic analysis identified upregulation of the expression of E2F targets, *KRAS* and TNF-alpha signaling, and epithelial-mesenchymal transition pathways in carcinomas compared to adenomas and normal tissues. A molecular classification model based on carcinoma-specific genes clearly separated carcinomas from adenomas and normal tissues, the clinical utility of which was demonstrated in two patients with uncertain malignant potential. A deeper analysis of gene expression and functional prediction suggested that Wilms tumor 1 (*WT1*) is a potential biomarker for *CDC73*-mutant parathyroid carcinoma, which was further validated through immunohistochemistry. Overall, our study revealed the genomic and transcriptomic profiles of parathyroid carcinoma and may help direct future precision diagnostic and therapeutic improvements.

## Parathyroid cancer: Genetic profiling points to precision diagnosis

Detailed genetic profiling of rare parathyroid gland cancer reveals the molecular mechanisms of tumor growth and possible diagnosis markers. Cancers of the parathyroid glands, found behind the thyroid in the neck, and essential for maintaining blood calcium levels, progress slowly but are often fatal, and complete cures are rare due to the lack of effective therapies. Sangwoo Kim, Yumie Rhee and co-workers at Yonsei University College of Medicine in Seoul, South Korea, conducted genomic and transcriptomic sequencing of 50 cancerous and healthy parathyroid tissues. They found very distinct mutations and expression patterns in cancerous tissue: genes linked to cell invasion and migration were upregulated, while known tumor-suppressing genes were downregulated. They also observed overexpression of the gene *WT1*, which is easily identified in most pathology laboratories and could act as a diagnostic marker for distinguishing malignant from benign parathyroid tumors.

## Introduction

Parathyroid carcinoma is a rare malignancy, occurring in <1% to 5% of patients with primary hyperparathyroidism^1–3^. Parathyroid carcinoma is characterized by progressive refractory hypercalcemia and concomitant skeletal and renal diseases, resulting in a 5-year survival rate of 40–86%^1,4^. Although progression is generally slow, a complete cure is rarely achieved after recurrence because of the lack of effective therapeutic intervention^4^. To date, the efficacy of systemic chemotherapy and local adjuvant radiotherapy has not been proven in the management of parathyroid carcinoma, leading to surgical resection as the only treatment option with proven benefits for survival and recurrence^5^. In particular, distinguishing between atypical parathyroid tumors (known as parathyroid neoplasms of uncertain malignant potential) and parathyroid carcinoma remains a major problem because of the ambiguous shared features observed through histological examination as well as technical difficulties in performing and interpreting relevant tests (e.g., parafibromin immunohistochemistry)^6,7^. These limitations in diagnosis and treatment have resulted in an urgent requirement for better therapeutic targets and biomarkers, as provided by genome- and transcriptome-level investigations.

For many other cancers, profiling of genomic characteristics has been attempted for parathyroid carcinoma to identify solutions for the current unmet clinical needs^4,8^. A major achievement is the identification of driver mutations in the tumor suppressor gene cell division cycle 73 (*CDC73*, previously known as *HPRT2*) encoding parafibromin, which accounts for ~40% of cases^9,10^. The biallelic inactivation of *CDC73* via the “two-hit” process (a germline predisposing mutation as the first hit and a somatic truncating mutation as the second) has also been reported^11^. Other genomic variants, such as somatic mutations in *MEN1* and *TP53*^12^ and somatic copy number alterations^8,13^, have been observed; however, the driving power of most mutations has remained provisional, mainly owing to the small cohort size and the lack of reproducible studies. Moreover, the transcriptomic characteristics of parathyroid carcinoma, such as cancer-specific gene expression and genetic and pathway-level activity, remain unexplored. Therefore, the discovery of genomic markers and the establishment of molecular classification models have proven challenging. The main difficulties lie in the rarity and lack of high-quality tissues from subjects with different conditions (i.e., malignant, benign, and normal) within a cohort. We expect that integrated analysis of genomic and transcriptomic data with clinical information will advance our understanding of parathyroid carcinoma and its translational usage.

Here, we analyzed the genomic and transcriptomic profiles of 50 parathyroid specimens that included parathyroid carcinoma and matched control samples (adenoma, normal tissues, and blood). This enabled us to profile the fundamental characteristics of genomic variations and gene expression, including germline and somatic mutations, copy number variations, allelic imbalances, differentially expressed genes and pathways, and molecular classification models. In addition, their potential use in precision diagnostics is discussed. We anticipate that our study will provide a foundation for the development of precision medicine strategies for parathyroid carcinoma, as for many other cancers.

## Materials and methods

### Sample acquisition

**Fig. 1:**
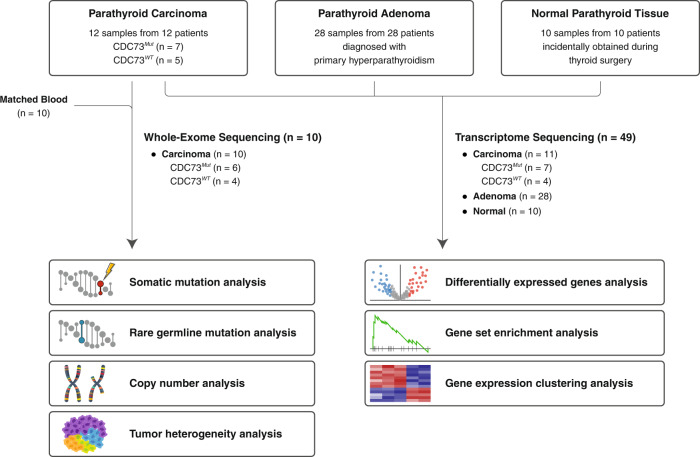
Schematic overview of the study workflow. This schematic summarizes the sample collection and data generation in this study. DNA from 10 carcinomas and their matched normal blood samples were sequenced for genomic profiling, and RNA from 11 carcinomas, 28 adenomas, and 10 normal parathyroid tissues were sequenced for transcriptomic profiling.

A total of 50 parathyroid specimens (28 adenomas, 12 carcinomas, and 10 normal parathyroid tissues) from 50 individuals (mean age, 52 years; women, *n* = 41) collected between 2015 and 2019 at Severance Hospital, Yonsei University Health System, Seoul, Republic of Korea, were analyzed in this study (Fig. 1, Supplementary Table 1). Adenoma and carcinoma samples were obtained during parathyroidectomy for primary hyperparathyroidism. Parathyroid tissues incidentally obtained from thyroidectomy for benign thyroid diseases or nonmetastatic thyroid cancer were collected and labeled normal parathyroid tissue, ascertained through gross pathology and biochemical features. In addition to processing samples for formalin-fixed paraffin-embedded (FFPE) for diagnosis at the pathology department, residual samples (roughly one-quarter of the sample size) were submerged in RNA preservative (RNA*later*, Invitrogen) immediately after excision in the operating room and then stored at –80 °C in the parathyroid tissue bank until thawing for RNA extraction.

Total RNA was isolated using a commercial kit (RNeasy Mini Kit, Qiagen, Seoul, South Korea) with RNA-Bee reagent (AMSbio) according to the manufacturer’s instructions. RNA concentration was assessed using a Qubit Fluorometer (Thermo Fisher), and RNA integrity was determined using a 4200Tapestation (Agilent). RNA at a concentration of ≥3 ng/µL was considered adequate for gene expression, and its quality was deemed acceptable when the DV200 value was ≥70%.

For parathyroid carcinoma samples, germline DNA from blood samples was obtained for the investigation of germline mutations, with matched FFPE-derived tumor DNA in ten subjects. Written consent was obtained from all subjects prior to surgery for the secondary use of surgical specimens for research purposes with proper deidentification, as per institutional protocols. Clinicopathological diagnoses of all tumor tissues were reviewed by two dedicated pathologists (SKK and GJK) who had >5 years of experience per the recent World Health Organization classification of tumors of the parathyroid gland^14^, with adjudication by multidisciplinary reviewers (YR, NH, and JJJ) comprising endocrinologists and endocrine surgeons when the pathological diagnosis was discordant or unclear prior to the analysis. This study was approved by the institutional review board of Severance Hospital (No. 4-2019-1018; Seoul, Republic of Korea).

### Generation of sequencing data

For accurate genomic analysis, whole-exome sequencing (WES) with a target depth of 200X was performed on 10 carcinoma samples, and a total of 10 WES data points with the same target depth were also generated from matched blood samples obtained with the consent of the patients. WES raw reads were aligned to the GRCh38 reference genome using a BWA-MEM (v0.7.17-r1188)^15^ aligner, and preprocessing was completed by employing MarkDuplicates and FixMateInformation included in GATK (v4.0.1.1)^16^.

Transcriptome sequencing was performed on 49 specimens, consisting of 28 adenomas, 11 carcinomas, and 10 normal parathyroid tissues after excluding one carcinoma sample that failed to pass the quality check (QC). Of these, 11 samples (8 adenomas and 3 carcinomas) were sequenced using the *Illumina Total RNA Sequencing* library, and the remaining 38 samples (20 adenomas, 8 carcinomas, and 10 normal tissues) were sequenced using the *SureSelectXT RNA Direct* library to minimize loss owing to QC failure (Supplementary Fig. 3a). All RNA sequencing data were aligned to the genome index generated from the GRCh38 reference genome and GENCODE (v33) annotation using the STAR2 (v2.7.8a)^17^ aligner. After alignment, to limit the target regions of the two sequencing libraries equally, all reads aligned to the regions not targeted by *SureSelectXT RNA Direct* were excluded from the *Illumina Total RNA Sequencing* data. Finally, RNA data preprocessing was completed using MarkDuplicates included in GATK (v4.0.1.1). The data generated in this study are publicly available in NCBI SRA at PRJNA854098.

### Identification of single nucleotide variants (SNVs) and insertion‒deletion mutations (INDELs)

Germline mutations of all specimens were identified using Strelka2 (v2.9.10)^18^. For the 10 carcinoma samples with WES data, the Strelka germline workflow was applied with multiple inputs of paired DNA. For the remaining 40 samples (2 carcinomas, 28 adenomas, and 10 normal tissues) without WES data, the Strelka RNA germline variant calling mode (*--rna*) was applied to the RNA-seq data.

Somatic mutations in the 10 carcinoma samples were called by GATK Mutect2 (v4.0.1.1) through the input of WES data with their matched normal data. When running Mutect2, the *--f1r2-tar-gz* argument was used to remove strand orientation bias artifacts, and further artifact filtering was applied using SOBDetector (v1.0.2)^19^ to the raw output of Mutect2. The mutation status of the *CDC73* gene, which was the focus of this study, was manually checked using IGV^20^ and is listed in Supplementary Table 1. If there were nonsynonymous SNVs or frameshift INDELs in the coding region of *CDC73*, whether they were somatic or germline, the sample was classified as *CDC73* mutant (*CDC73*^*Mut*^).

### Mutational signature analysis

Mutational signature analysis was performed using the *trinucleotideMatrix()* function of maftools (v.2.12.0)^21^, with the Mutect2 results described above. To retain only the variants from the major clone and minimize the risk of sequencing artifacts, a VAF > 0.05 filter was additionally applied to the somatic variant call set, as done in a previous study^22^. The APOBEC enrichment score was also obtained from the results of the *trinucleotideMatrix()* function. The score is the ratio of C > T mutations with the TCW motif, which is a result of APOBEC enzyme activity, to the background cytosines and TCWs. The calculation is as follows:

$$\frac{{n_{tCw} \times background_C}}{{n_C \times background_{TCW}}}$$

A one-sided Fisher’s exact test was performed for APOBEC enrichment, as described in a previous study^23^.

### Allele-specific copy number analysis

The allele-specific copy number was inferred by combining the relative depth information for each bin of the genomic region and the B-allele frequency information of the germline hetero SNP belonging to the corresponding bin (Supplementary Fig. 9). Germline hetero SNPs were extracted from the results of haplotype callers (after excluding homo SNPs), and the subsequent process was performed using two tools, Sequenza (v.3.0.0)^24^ and PureCN (v.2.1.2)^25^, for cross-validation.

### Calculation of intratumor VAF

The following corrections were applied to the observed allelic counts to obtain VAFs that were expected to be entirely derived from tumor cells. The observed number of reference alleles (REF) and alternate alleles (ALT) were deconvoluted as follows:

$$REF = REF_{normal} + REF_{tumor}$$

$$ALT = ALT_{normal} + ALT_{tumor}$$

and we defined the intratumor VAF ($VAF_{tumor}$) as follows:

$$VAF_{tumor} = \frac{{ALT_{tumor}}}{{REF_{tumor} + ALT_{tumor}}}$$

If the tumor purity (*p*) and absolute allele-specific copy number (*CN*) of tumors are known, $REF_{tumor}$ and $ALT_{tumor}$ on the germline variant locus can be calculated as follows:

$$REF_{tumor} = REF \times \frac{{pCN_{tumor}^{REF}}}{{\left( {1 - p} \right)CN_{normal}^{REF} + pCN_{tumor}^{REF}}} = REF \times \frac{{pCN_{tumor}^{REF}}}{{\left( {1 - p} \right) + pCN_{tumor}^{REF}}}$$

$$ALT_{tumor} = ALT \times \frac{{pCN_{tumor}^{ALT}}}{{\left( {1 - p} \right)CN_{normal}^{ALT} + pCN_{tumor}^{ALT}}} = ALT \times \frac{{pCN_{tumor}^{ALT}}}{{\left( {1 - p} \right) + pCN_{tumor}^{ALT}}}$$

where $CN_{tumor}^{REF}$ denotes the REF-side copy number of the tumor, $CN_{tumor}^{ALT}$ denotes the ALT-side copy number of the tumor, and $CN_{normal}^{REF}$ and $CN_{normal}^{ALT}$ are assumed to be 1. Tumor purity and absolute allele-specific copy number can be inferred using numerous tools; however, we used cross-validated values from Sequenza and PureCN in this study.

In the case of the somatic variant locus, REF alleles derived from ALT-side haploid normal cells were also observed.

$$REF_{tumor} = REF \times \frac{{pCN_{tumor}^{REF}}}{{\left( {1 - p} \right)CN_{normal}^{REF} + (1 - p)CN_{normal}^{ALT} + pCN_{tumor}^{REF}}} = REF \times \frac{{pCN_{tumor}^{REF}}}{{2(1 - p) + pCN_{tumor}^{REF}}}$$

$CN_{normal}^{ALT}$ of the somatic variant locus is always zero, as the somatic ALT alleles are derived only from tumor cells.

$$ALT_{tumor} = ALT$$

The same calculation can be applied to the RNA allelic count if the following conditions are satisfied.

1. The gene containing this locus was not a differentially expressed gene (DEG) in the cohort.
  (i.e., normal and tumor cells usually express the gene at the same rate).
2. The number of expressed transcripts is proportional to the copy number of the template.

As *CDC73* was not found to be a significant DEG in the cohort, these conditions were satisfied for variants within *CDC73* (Supplementary Fig. 7).

### Identification of carcinoma- and adenoma-specific DEGs

From the preprocessed RNA BAM file, the number of reads per gene region was counted using FeatureCount (v.2.0.1)^26^. The raw count data were input to DESeq2 (v.1.30.1)^27^ along with group labels of adenoma and carcinoma and sequencing batch labels (Supplementary Table 1). We first confirmed that there was no sequencing depth related bias across the samples by checking FPKM distributions of each group (Supplementary Fig. 3b). These data were converted to a normalized value of the variance stabilizing transformation (VST) implemented in DESeq2. Through principal component analysis (PCA) of these VST values, it was confirmed that the batched effect was successfully removed throughout the entire set of VST values (Supplementary Fig. 3c, d).

A 2-axis DEG analysis was performed by applying the *vsFourWay()* function of ViDGER (v.1.10.0)^28^ to the VST values. DEGs were selected by applying cutoffs of |log2 | > 1 and padj < 0.01 compared to the corresponding VST of the normal group (Fig. 3a). The combination of |log2 | > 2 and padj < 0.05 was also applied to select DEGs at various cutoffs (Supplementary Table 2, Supplementary Fig. 11).

The same method was applied to the carcinoma and normal samples to identify DEGs of *CDC73*^*Mut*^ and *CDC73*^*WT*^ compared to the normal cohort (Supplementary Table 4, Supplementary Fig. 12). Classification of *CDC73*^*Mut*^ and *CDC73*^*WT*^ carcinomas was established according to the Strelka germline calls and Mutect2 calls (Supplementary Table 1).

### Gene set enrichment analysis

The VST values obtained from DESeq2 were converted into the input format required for GSEA (v.4.1.0)^29^. The phenotype labels defining the groups to be compared for each analysis are provided in Supplementary Table 1. “*Hallmark gene sets (H)*” and “*gene_set*” were selected for the “gene sets database” and “permutation type” parameters, respectively. The ENSEMBL gene IDs within the VST table were altered to HGNC gene symbols using an annotation file hosted on the GSEA-MSigDB file server. Parameters other than those specified were configured according to the GSEA user guidelines.

### Hierarchical clustering with DEGs

The VST values of all genes (*n* = 19,504) and carcinoma-specific DEGs obtained through 2-axis DEG analysis were collected for molecular classification. Hierarchical clustering was performed using the *pheatmap()* function of the R package pheatmap (v.1.0.12) according to the VST values of the DEGs. DEGs were tested with a total of four sets with a combination of two *p*-values (*p* < 0.01, *p* < 0.05) and two log2-fold change cutoffs (lfc > 1, lfc > 2).

### Immunohistochemistry (IHC)

Herein, 28 adenoma and 10 carcinoma tissues were collected for immunohistochemical analysis. Formalin-fixed, paraffin-embedded tissue blocks were cut into 4-μm sections. Immunohistochemical staining was performed using a Ventana XT automated stainer (Ventana Medical System, Tucson, AZ, USA) with an antibody against Wilms tumor 1 (WT1) (clone 6F-H2, 1:100; Cell Marque, Rocklin, CA, USA) according to the manufacturer’s instructions. The negative control samples were processed without the primary antibody. The positive control tissue was used according to the manufacturer’s recommendations. IHC staining was evaluated using light microscopy (Supplementary Fig. 14). Nuclear expression was semiquantitatively evaluated using stained slides as previously described^30^. WT1 expression was graded according to the intensity of nuclear expression as weak, moderate, or strong. Tissues exhibiting WT1 expression in <5% of the tumor cells of any intensity grade or those with weak intensity were regarded as negative, while tissues showing moderate to strong intensities in >5% of the tumor cells were regarded as positive for WT1 expression.

### Identification of WT1 transcript

The identification of transcripts was confirmed by checking the exon usage of *WT1* using DEXSeq (v. 1.44.0). The same read count table generated by FeatureCount as described above was used again as input. With the python script *dexseq_prepare_annotation.py* provided with the DEXSeq package, the FeatureCount files were converted to the proper input format for the DEXSeq. Then, the DEXSeqDataSetFromHTSeq() function of the DEXSeq R package was used to generate a *DEXSeqDataSet* object. The relative exon usage was checked based on the transcript reference of Ensembl 108.

## Results

### Clinical and biochemical characteristics of parathyroid carcinoma

In total, 50 thyroid tissues were collected from three groups, 12 parathyroid carcinomas, 28 parathyroid adenomas, and 10 normal parathyroid tissues, for genomic and transcriptomic profiling (Fig. 1). The detailed protocols and quality control procedures are described in the Materials and Methods section. For genomic profiling, WES of carcinoma tissues with matching blood samples was conducted. For transcriptomic profiling, RNA sequencing was conducted for all three groups, and the resulting data were used for gene and gene set-level analyses.

We first analyzed the clinical and biochemical characteristics of parathyroid tumors (Table 1). While the normal group individuals were the youngest (mean age = 38.4), the carcinoma group individuals were younger (mean age = 42.2, *p* = 0.0009, Mann‒Whitney test) than the adenoma group individuals (mean age = 61.3) (Supplementary Fig. 1), representing a prevalent genetic risk factor (i.e., loss of heterozygosity at the *CDC73* locus^31^). We found a female predominance in all three groups (67–89%), without statistically significant group-specific differences. Patients with parathyroid adenoma and carcinoma exhibited higher preoperative parathyroid hormone (PTH) and serum calcium levels than normal individuals, both of which were highest in carcinoma patients. Likewise, phosphate levels were distinctive among all three groups, while they were the lowest in carcinoma patients. Clinical genetic tests (targeted sequencing of blood) identified germline mutations in *CDC73* in six out of 12 carcinoma patients, showing a compatible frequency with those of previous reports (41–61.8%)^10,32^. Among the 12 patients with parathyroid carcinoma, three had distant metastasis, and two had local recurrence during follow-up. These findings are consistent with the known clinical characteristics and prognosis of parathyroid carcinoma^1^.

**Table 1:** Clinical characteristics of the study subjects.

	Normal (*n* = 10)	Adenoma (*n* = 28)	Carcinoma (*n* = 12)	*P*-value
Age, yr	35 [31–45]	64 [56–69]*	43 [25–58]*	<0.001
Women, *n* (%)	8 (80)	25 (89)	8 (67)	0.164
Preoperative PTH, pg/mL	35.0 [25.9-45.2]	114.5 [91.8-218.9]*	214.6 [137.9-347.4]*^†^	<0.001
Corrected calcium, mg/dL	9.7 [9.4–9.9]	10.9 [10.5-11.6]*	12.3 [11.7-13.6]*^†^	<0.001
Inorganic phosphorus, mg/dL	3.9 ± 0.5	2.9 ± 0.5*	2.4 ± 0.6*^†^	<0.001
24 hr urine calcium, mg/dL	N/A	267 ± 144 (*n* = 24)	364 ± 94 (*n* = 5)	0.161

*p < 0.05 vs. normal; ^†^*p* < 0.05 vs. adenoma.

### Genomic profiles of parathyroid carcinoma

**Fig. 2:**
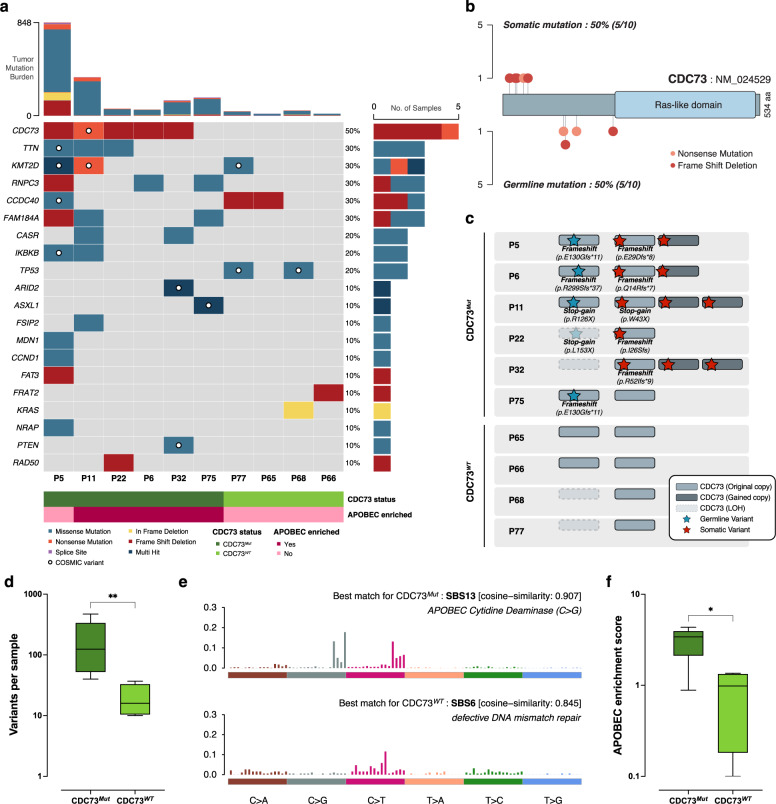
Genomic profiles of parathyroid carcinoma. **a** Somatic variants found in parathyroid carcinoma samples. The somatic status of each variant was determined by confirming the absence of the corresponding variant in matched normal data, and only mutated genes found in three or more samples or genes that have been reported in other PTC studies were plotted. If there was a truncating mutation in the *CDC73* coding region regardless of whether it was somatic or germline, the sample was classified as *CDC73*^*Mut*^; otherwise, the sample was classified as *CDC73*^*WT*^. **b** Genomic positions of *CDC73* mutations in the whole carcinoma cohort. All *CDC73* mutations were truncating mutations, and somatic mutations were found upstream of the gene. **c** Allele-specific copy number status of the genomic region including *CDC73*. Somatic mutated copies tended to have copy number gain. **d** The number of variants per sample found in the *CDC73*^*Mut*^ and *CDC73*^*WT*^ groups. Significance between the two groups was confirmed by the Mann‒Whitney test (*p* = 0.0095). **e** Mutational signatures of parathyroid carcinoma. SBS13 was revealed to be the major signature of the *CDC73*^*Mut*^ group, whereas SBS6 was found in the *CDC73*^*WT*^ group. **f** APOBEC enrichment score of the *CDC73*^*Mut*^ and *CDC73*^*WT*^ groups. The score was significantly high in *CDC73*^*Mut*^ (*p* = 0.0381, Mann‒Whitney test).

To investigate the profile of genomic variations in parathyroid carcinoma, we identified somatic mutations in 10 carcinoma samples (out of 12), wherein matching blood samples were available (Fig. 2a, see Methods). The number of nonsynonymous single nucleotide variants (SNVs) and insertion‒deletions (indels) ranged from 18–848, with a median of 59, which corresponded to 1.18 mutations per megabase. Except for one sample with an exceptionally high mutation count (P5), all carcinomas had relatively lower mutation counts than other cancers^33,34^.

First, we examined the mutation patterns of *CDC73*. Six patients (P5, P6, P11, P22, P32, and P75) who had either germline or somatic truncating mutations (four nonsense SNVs and seven frameshift deletions) were considered *CDC73*-mutant (*CDC73*^*Mut*^). We found that the genomic loci of germline and somatic mutations were clearly separated (Fig. 2b); germline mutations were located upstream of the Ras-like domain, which is crucial for interaction with PAF1 (polymerase associated factor 1) and chromatin^35^, whereas somatic mutations were clustered at exon 1–2, suggesting a complete loss of transcript. Four of the six patients (P5, P6, P11, and P22) showed apparent two-hit mutations that caused biallelic inactivation of *CDC73*, known as Knudson’s two-hit hypothesis (i.e., one germline predisposition and one acquired somatic mutation). One patient (P32) had a somatic frameshift mutation only (CDC73 p.R52Ifs*9), but it was accompanied by a complete loss of the wild-type allele due to loss of heterozygosity (LOH), resulting in biallelic inactivation (Fig. 2c). One patient (P75), who only had a germline truncating mutation (CDC73 p.E130Gfs*11), showed borderline to early pathological classification, indicating a chance for a subclonal somatic mutation. Overall, the mutation patterns in all six patients showed truncating *CDC73*-directed consequent biallelic inactivation, opposing the existence of secondary hits in genes other than *CDC73*.

Next, we assessed the mutation profiles of four patients (P65, P66, P68, and P77) with wild-type *CDC73* (*CDC73*^*WT*^). We found that the number of variants was lower in *CDC73*^*WT*^ (median = 29.0) than in *CDC73*^*Mut*^ (median = 152.5; *p* = 0.0095, Mann‒Whitney test) patients (Fig. 2d), indicating relatively higher genomic integrity^36,37^. Despite the limited sample numbers, we observed a few recurrent mutations within *CDC73*^*WT*^ patients. Somatic missense mutations in *TP53* were observed in two *CDC73*^*WT*^ patients (*TP53 p.C3F* in P68 and *TP53 p.H61R* in P77), both of which have been previously reported in other cancers and are expected to be deleterious. Although not statistically significant (*p* = 0.1333, Fisher’s exact test), this may imply the confinement of *TP53* mutations in *CDC73*-independent parathyroid carcinoma. Similarly, of the three somatic mutations in *CCDC40* (coiled-coil domain containing 40), two truncation frameshift mutations (CCDC40 p.K970Nfs*51 and CCDC40 p.G987Rfs*96) were found in *CDC73*^*WT*^ patients (P65 and P77). The *CCDC40* mutation is best known as the major cause of primary ciliary dyskinesia, but its association with cancer has not yet been reported. Other mutations in *KMT2D*, *KRAS* (in-frame deletion), and *FRAT2* (FRAT regulator of WNT signaling pathway 2) are potentially associated with the oncogenesis of parathyroid carcinoma; however, the evidence remains limited.

Mutational signature analysis revealed two distinct signatures for each carcinoma group: SBS13 (single base substitution signature 13) for *CDC73*^*Mut*^ and SBS6 for *CDC73*^*WT*^ (Fig. 2e**, see Methods**). SBS1 was additionally found in the *CDC73*^*Mut*^ group, which was later confirmed to be the exclusive signature of P5 and not present in every *CDC73*^*Mut*^ sample (Supplementary Fig. 2). SBS13, the major signature found in the *CDC73*^*Mut*^ group, is known for its association with activated APOBEC cytidine deaminase^38,39^ and has been proposed as a marker for immunotherapy and targeted therapy^40–42^. Additional analysis of APOBEC enrichment also confirmed significantly high APOBEC relevance in the *CDC73*^*Mut*^ group (Fig. 2f), which could be another clue to the different mechanisms of tumor progression in *CDC73*^*Mut*^ and *CDC73*^*WT*^ carcinomas.

### Transcriptomic analysis of parathyroid carcinoma

**Fig. 3:**
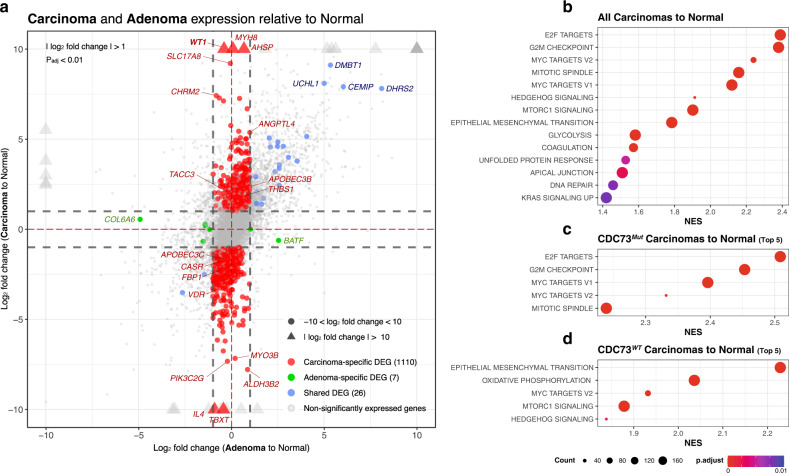
Transcriptomic analysis of parathyroid carcinoma and adenoma compared to normal tissue. **a** Two-axis DEG analysis of parathyroid carcinoma and adenoma compared to normal parathyroid tissue. *p*_adj_ < 0.01 and |log_2_-fold change | > 1 were used as cutoffs for this analysis. The *X*-axis represents the DEGs of adenoma to normal, and the *Y*-axis represents the DEGs of carcinoma to normal. Red dots represent carcinoma-specific DEGs, green dots represent adenoma-specific DEGs, and blue dots represent common DEGs of adenoma and carcinoma. **b**–**d** GSEA results of all carcinomas, *CDC73*^*Mut*^ carcinomas and *CDC73*^*WT*^ carcinomas compared to the expression of normal parathyroid. Enrichment analysis was performed on the H collection (hallmark gene sets) of MSigDB. Each term is shown without ‘HALLMARK’.

Using RNA sequencing data of 49 tissues (11 carcinomas, 7 *CDC73*^*Mut*^ and 4 *CDC73*^*WT*^; 28 adenomas; and 10 normal parathyroid tissues), DEGs (see Methods for criteria) were analyzed for two conditions: carcinoma vs. normal and adenoma vs. normal (Fig. 3a). We found that the overall gene expression profiles were highly conserved in adenomas, showing a strong correlation with those of normal parathyroid glands (Pearson’s *r* = 0.982) (Supplementary Fig. 4). In contrast, gene expression in carcinomas deviated substantially (Pearson’s *r* = 0.943). Therefore, the number of DEGs was larger in carcinoma tissues (*n* = 1,136) than in adenoma tissues (*n* = 33), 26 of which were differentially expressed in both. Consequently, 1,110 carcinoma- and seven adenoma-specific DEGs were identified (Fig. 3a red and green dots, respectively; see Supplementary Table 2 for a full list of DEGs).

Pathway-level analysis revealed the enrichment of many cancer hallmark pathways in carcinoma (Fig. 3b, Supplementary Table 3, Supplementary Fig. 5). In particular, pathways involved in E2F targets, G2M checkpoint, glycolysis, Myc targets, and epithelial-mesenchymal transition (EMT) were upregulated compared to normal samples (adjusted *p*-value < 0.01 and FDR < 0.25, see Methods). Mild upregulation of G2M and Myc target pathways was also observed in adenoma, but *KRAS* signaling and TNF-alpha signaling were downregulated, in contrast to carcinoma (*p* < 0.003). In the GO enrichment results of adenoma-DEGs, including even subtle DEGs of 1.5-fold change to normal, upregulation of genes belonging to cell growth or neuronal development was observed (Supplementary Fig. 6).

Further assessment revealed differences in pathway activation between *CDC73*^*Mut*^ and *CDC73*^*WT*^ (Supplementary Table 5, Supplementary Fig. 5). The upregulation of E2F targets was observed more significantly in *CDC73*^*Mut*^, which might suggest an activated DNA damage response against the higher mutation burden^43^ (Fig. 3c). While activation of E2F and Myc targets, mTORC1 and Hedgehog signaling were commonly observed, *CDC73*^*WT*^ parathyroid carcinoma showed stronger activation of EMT and oxidative phosphorylation (Fig. 3d). EMT activation in tumor progression is well known to be significantly involved in tumorigenesis and angiogenesis. Moreover, the combination of upregulated oxidative phosphorylation can be strong proof of metastasis^44,45^ and, more specifically, the hybrid E/M phenotype^46–48^. Indeed, one of the patients in the *CDC73*^*WT*^ cohort showed multiple metastases after sample preparation. Although no signs of metastasis were found in other *CDC73*^*WT*^ patients, the possibility of metastasis cannot be ruled out considering this result.

### Allelic imbalance and allele-specific expression of *CDC73*

**Fig. 4:**
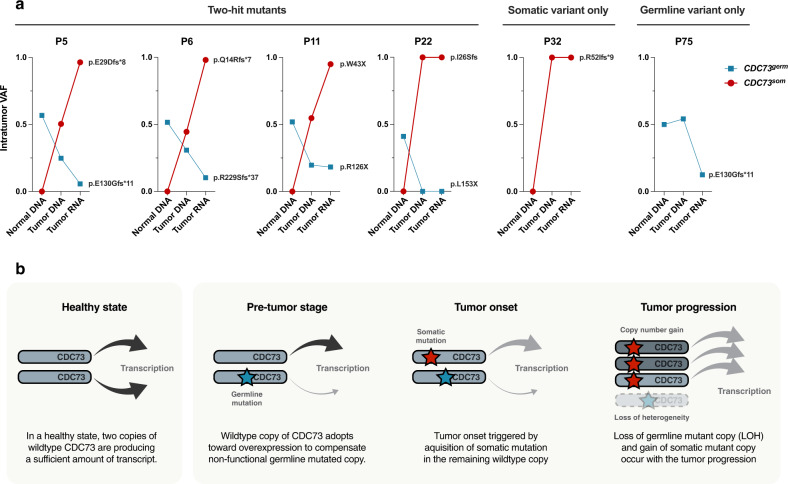
Allelic imbalance and allele-specific expression of *CDC73*. **a** Allele frequencies of *CDC73* mutations traced through normal DNA, tumor DNA, and tumor RNA and corrected to the values expected to be entirely derived from tumor cells. In the two-hit mutant group, the somatically mutated copy tended to have a higher copy number than the copy with the germline mutation. Furthermore, the expression of somatically mutated copies is commonly upregulated, and as in the case of P75, the expression of the first-hit copy is suppressed even though there is no second hit. **b** Hypothesis for the onset and progression of parathyroid carcinoma in terms of *CDC73* mutation status. The size of the arrows indicates the transcription rate, and the light gray arrow indicates nonfunctional transcription.

As shown earlier, two-hit mutations in *CDC73* result in two separable alleles: one allele that harbors germline variants (referred to as *CDC73*^*Germ*^) and the other that acquires somatic mutations (*CDC73*^*Som*^). Combined analysis of genomic and transcriptomic profiles enabled the inspection of DNA- and RNA-level imbalances, including allele-specific copy number alterations (CNAs) and expression biases between the two alleles. We detected frequent (70%, 7/10) allele-specific CNAs at 1q31.2, which included the genetic lesion of *CDC73* (Supplementary Fig. 8). Notably, all four samples with *CDC73* two-hit mutations (P5, P6, P11, and P22) showed elevated intratumor variant allele frequency (*VAF*_*tumor*_) of somatic variants, while the *VAF*_*tumor*_ of germline variants decreased in tumor DNA, suggesting copy number gains in *CDC73*^*Som*^ and/or losses in *CDC73*^*Germ*^ (Fig. 4a, Supplementary Fig. 9, see Methods). Likewise, as described previously (Fig. 2c), the patient with *CDC73* LOH (P32) retained *CDC73*^*Som*^ only. Therefore, all five patients with *CDC73* somatic mutations showed a relative gain in *CDC73*^*Som*^.

Further analysis revealed additional bias at the transcriptional level. Allele-specific RNA-seq analysis (Fig. 4a, Supplementary Fig. 10, see Methods) showed 2.3- to 7.9-fold higher gene expression in *CDC73*^*Som*^ than expected (from the DNA-level allele frequency) in the four patients with *CDC73* two-hit mutations. These results indicate that the allelic gain in *CDC73*^*Som*^ is not only retained but further intensified at the transcription level owing to allele-specific expression. Likewise, we found that the gene expression of *CDC73*^*Germ*^ in P75, which harbored the *CDC73*^*Germ*^ mutation only, was substantially lower than expected, also supporting the higher allele-specific expression in *CDC73*^*Som*^ in all six *CDC73*^*Mut*^ patients.

Based on the results, we suggest a plausible model that explains the duplex preference (genomic and transcriptomic) of *CDC73*^*Som*^ over *CDC73*^*Germ*^ (Fig. 4b). Born with one inactivated allele (*CDC73*^*Germ*^), the other intact allele (*CDC73*^*Wt*^) solely takes the designated role of the gene, such as cellular homeostasis and tumor suppression. This leads to a more active use of *CDC73*^*Wt*^ before tumorigenesis, which can be achieved by either deteriorating *CDC73*^*Germ*^ (e.g., copy loss or transcriptional suppression) or positively selecting *CDC73*^*Wt*^ (e.g., copy gain or transcriptional activation). At the time of tumorigenesis, the second hit (somatic truncation mutations) converts *CDC73*^*Wt*^ to *CDC73*^*Som*^, maintaining the genomic and transcriptomic preference over *CDC73*^*Germ*^, with further selective advantages acquired during tumor progression. Overall, our model explains the allelic imbalance and allele-specific expression in *CDC73* based on the functional compensation for the haploinsufficiency of *CDC73* caused by germline truncating mutations, as reported several times in other studies^49,50^.

### Molecular classification of parathyroid carcinoma and adenoma

**Fig. 5:**
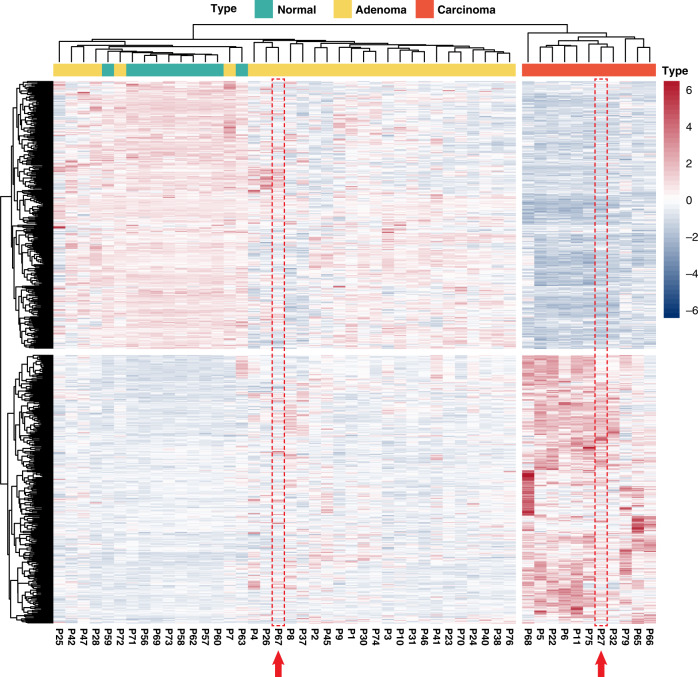
Molecular classification of parathyroid carcinoma and adenoma. Hierarchical clustering result using 597 carcinoma-specific DEGs (red dots in Supplementary Fig. 11g). All carcinomas were well-divided and clustered from adenomas and normal tissues, suggesting that the feature genes are sufficient to classify carcinomas.

Based on the transcription profiles identified using RNA-seq, we constructed a molecular classification model for parathyroid carcinoma from adenoma and normal parathyroid glands. A total of 597 genes with strong carcinoma specificity were selected from the DEG sets by applying the most stringent filtration method (Supplementary Fig. 11g) and were used for hierarchical clustering of 49 samples (11 carcinomas, 28 adenomas, and 10 normal parathyroid tissues). In the initial clustering without any training or optimization, we found that all carcinoma samples were clearly separated from non-carcinoma samples (Fig. 5), indicating intrinsic differences in the molecular characteristics that are present in the gene set.

The clinical utility of the molecular classification was shown in two patients: P27 and P67. Both patients were initially diagnosed with atypical parathyroid neoplasm with uncertain malignant potential but were separately clustered; P27 was clustered with carcinoma, and P67 was clustered with adenoma (Fig. 5 red arrows). Further prospective follow-up identified clinical recurrence in P27, whereas no signs of pathological progression, including typical capsular or vascular invasion, were observed in P67. Further cohort-level studies are required to validate the utility of molecular classification in cases with uncertain malignant potential, which take place in 0.5–5% of parathyroid tumors^1,4^.

We also found that the non-carcinoma group was divided into two subgroups. Therefore, we checked whether they are biologically distinctive from each other. However, the adenomas in Group 1 and Group 2 did not show significant differences either in their transcriptome or clinicopathology (Supplementary Fig. 13, Supplementary Table 6).

### *WT1* as a potential marker for *CDC73*-mutant parathyroid carcinoma

Using whole transcriptome sequencing data, we searched for a possible single-gene marker for parathyroid carcinoma. Among the genes with carcinoma-specific expression (Fig. 4a red dots), we focused on Wilms tumor 1 (*WT1*) based on its functional relatedness with *CDC73*; *WT1* is known to directly repress *CDC73* and induce *MYC* and *BCL-2* to promote cell proliferation and tumorigenesis^51^. In addition, *WT1* has been considered a single molecular biomarker for multiple cancers due to its consistent upregulation in tumors^52–54^.

**Fig. 6:**
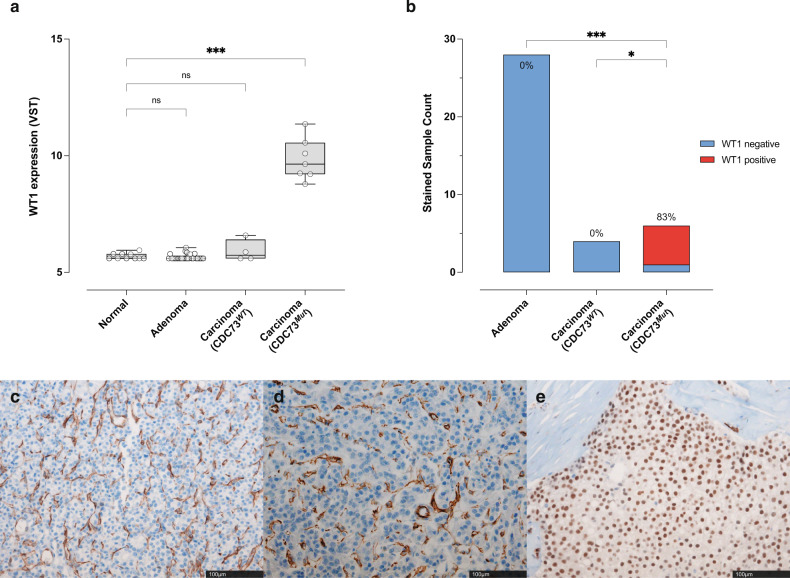
Differential expression of *WT1* in *CDC73*^*Mut*^ carcinomas. **a** Groupwise comparison of *WT1* expression. In *CDC73*^*Mut*^ carcinomas, significant overexpression of *WT1* was observed (*p* < 0.0001 in Kruskal‒Wallis test). **b** IHC-stained sample counts. WT1 positivity was found only in the *CDC73*^*Mut*^ sample group, and none of the adenomas and normal samples were found to be WT1 positive. Fisher’s exact test was performed with the number of WT1-negative versus WT1-positive samples in each group. The number at the top of the bar indicates a positive ratio in each group. **c** WT1-negative adenoma sample (P2). **d** WT1-negative *CDC73*^*WT*^ carcinoma sample (P65). **e** WT1-positive *CDC73*^*Mut*^ carcinoma sample (P6).

We tested the feasibility of using *WT1* as a single-gene biomarker for *CDC73*-mutant parathyroid carcinoma. We found that the overexpression of *WT1* in carcinoma was specific to *CDC73*^*Mut*^ patients but not present in *CDC73*^*WT*^ patients (Fig. 6a). In addition, immunohistochemical (IHC) staining of WT1 in 38 parathyroid tissues (28 adenomas, 4 *CDC73*^*WT*^ carcinomas, and 6 *CDC73*^*Mut*^ carcinomas) confirmed the presence of *CDC73*^*Mut*^–specific *WT1* in parathyroid cancer tissues (Fig. 6b). That is, neither adenomas (Fig. 6c) nor *CDC73*^*WT*^ carcinomas (Fig. 6d) were stained with the WT1 antibody, but five *CDC73*^*Mut*^ carcinomas (out of 6, Fig. 6e) showed positive staining (Supplementary Fig. 14 for more IHC staining images). Since specific splicing alternatives of *WT1* are known to be associated with certain diseases, we further checked the transcript type of *WT1* with DEXSeq^55^, and we confirmed that the overexpressed *WT1* in the *CDC73*^*Mut*^ group was a canonical transcript (ESNT00000452863.10, Ensembl 108, Supplementary Fig. 15). We anticipate that these results will provide a basis for the future development of a clinical test for a faster, cheaper, and more accurate diagnosis of parathyroid cancer and its mutation status.

## Discussion

In this study, we conducted genomic and transcriptomic analyses of 50 parathyroid tissues composed of carcinomas, adenomas, and their matched controls. In the absence of whole-exome and transcriptome-scale studies, our study provides a foundation for mutation and gene expression profiles in parathyroid carcinoma, the assessment of which may lead to the identification of novel molecular characteristics. In particular, identification of allelic imbalance and allele-specific expression in *CDC73*, construction of molecular classification, and identification of WT1 as a potential biomarker are expected to advance the understanding of molecular mechanisms and more efficient diagnosis of parathyroid carcinoma.

Other findings on genomic mutations, differentially expressed genes, and pathways provide interesting clues to the molecular mechanisms involved in parathyroid carcinoma together with evidence from previous studies. First, we found recurrent germline mutations in *OGDHL* (oxoglutarate dehydrogenase L) specific to carcinoma patients (4/11, 36%) (Supplementary Fig. 16). Three of the four mutations have been reported in the COSMIC database as cancer-related variants, posing the possibility of a predisposition to parathyroid carcinoma. *OGDHL* overexpression and knockdown studies have shown that OGDHL inhibits cell growth and migration by inactivating ATK signaling^56^. Moreover, a recent study revealed that rare variants of *OGDHL* were significantly correlated with breast cancer in a Chinese cohort^57^, and its association with parathyroid carcinoma is also worth considering. Second, carcinoma-specific gene expression or repression suggests the underlying molecular mechanisms of tumor progression (Fig. 3a). Upregulation of the expression of *ANGPTL4* (angiopoietin like 4), *TACC3* (transforming acidic coiled-coil containing protein 3), and *THBS1* (thrombospondin 1), which have been reported in previous studies on parathyroid carcinoma^58–60^, was also observed in our study. Experimental evidence in multiple cancers or cell lines suggests associations between activation of the genes and cell invasion and migration via different mechanisms: *ANGPTL4* by regulating vascular permeability and angiogenesis^61^, *TACC3* by promoting G1/S transition and the Wnt/beta-catenin pathway^62^, and *THBS1* via interaction with tumor cell-bound CD47^63^. Likewise, downregulation of *FBP1* (fructose-1,6-bisphosphatase), vitamin D receptor (*VDR*), and calcium sensing receptor (*CASR*) was reproduced in our study and in previous reports^60,64,65^. Tumor-suppressing roles have been suggested for these genes in previous studies, including *FBP1* as an aerobic glycolysis inhibitor^66^ and *VDR* and *CASR* in negative regulatory feedback by calcium ions or calcitriol^64,65^. Third, the mutational etiology could be further investigated using gene expression. A previous study reported an enriched APOBEC mutational signature in parathyroid carcinoma^67^. In this regard, the carcinoma-specific upregulation of APOBEC3B and downregulation of APOBEC3C expression, which were found in our study, can provide strong support for cancer-associated etiology. Notably, an enriched APOBEC mutational signature along with high levels of APOBEC3B expression was associated with poor prognosis and potentially better response to immunotherapy in non-small cell lung cancer^41^. Whether the APOBEC mutational signature and altered expression of APOBEC3B have prognostic value in parathyroid carcinoma requires further investigation. Finally, we conducted immune profiling based on the transcriptomic data. We found that the fraction of immune cells was significantly lowered only in the *CDC73*^*Mut*^ group (Supplementary Fig. 17). Further in-depth studies are needed to elucidate the immune properties of carcinomas and adenomas, but the immune suppression in the *CDC73*^*Mut*^ group does not seem to be due to the T_reg_-mediated phenomenon, as the fraction of T_regs_ was also significantly lower.

Based on the comparative transcriptomic analysis between parathyroid carcinoma and adenoma samples, we found that there were no known shared damaging germline mutations between carcinomas and adenomas. Germline mutation of *CDC73* was exclusively found in carcinoma samples. In addition, no recurrent damaging germline mutations were found among adenomas. The gene expression pattern was significantly different in adenomas and carcinomas, showing only 26 shared DEGs, including *DMBT1*, *UCHL1*, *CEMIP*, and *DHRS2*. Although we were not able to perform additional sequencing to check further somatic variants or CNAs due to a lack of available adenoma samples at this point, our findings support the notion that parathyroid carcinoma may commonly arise *de novo* rather than evolving from adenoma. This notion has also been supported by prior studies showing clear distinctions in patterns of genomic and genetic alterations between parathyroid carcinoma and adenoma, similar to the findings in this study^68–70^. Clinically, the age of diagnosis of parathyroid carcinoma is ~10 years earlier than that of adenoma (mid-40s versus mid-50s)^1^, which also supports the potential of *de novo* onset of parathyroid cancer with distinctive genomic alterations rather than progression from adenomas. This needs to be further clarified in future studies.

As our study also aimed to discover biomarkers for better diagnosis, we focused on single genes that may separate carcinomas from adenomas and normal tissues. Our finding on *CDC73*^*Mut*^-specific overexpression of *WT1* provides a potential single-gene marker validated at the RNA and protein levels (RNA-seq and immunohistochemistry). An additional technical advantage of utilizing WT1 IHC staining in parathyroid tumors is that WT1 IHC staining is readily available in most pathology laboratories without difficulties in performance and interpretation. We expect that further studies will evaluate the use of *WT1* as a prognostic marker based on its association with poor survival and disease recurrence in multiple solid cancers^71^. In addition to *WT1*, we also found carcinoma-specific upregulation of *LGALS3* (galectin-3) and *UCHL1* (PGP9.5) (also upregulated in adenoma), which has been suggested as an IHC marker in parathyroid carcinoma^72,73^.

Despite all efforts, there are a few caveats in the interpretation of this study. First, two different RNA-seq technologies were applied to our samples, depending on the quantity and quality of the tissues; specifically, target-enriched mRNA sequencing was applied to the samples with lower quantity (see Methods). This is mainly because of the difficulty in securing a sufficient amount of fresh parathyroid tissue. Although we are aware of potential artifacts, such as the batch effect and applied strict normalization, there are some possible issues in the comparative analysis of gene expression. Second, although considered well-established computational techniques, inferring the exact copy number status and allele-specific expression from read depth and variant allele frequency is susceptible to external factors, such as read length and sample purity^74,75^. Further studies using single-cell sequencing or long-read sequencing data are needed to clarify this limitation. Finally, IHC staining data for parafibromin, PGP9.5, or galectin-3 were not available, although we performed WT1 IHC staining for study purposes. Comparative analysis of *WT1* with other IHC panels in future studies would strengthen our results.

We identified distinctive molecular characteristics of parathyroid carcinoma using comparative transcriptomic analysis of carcinoma, adenoma, and normal parathyroid gland samples. We anticipate that the mutational and transcriptional profiles, genes with phenotype-specific expression, allele-specific biases, and potential single-gene markers will provide novel insight for future research to improve diagnosis and individualized therapeutic strategies, which have been performed in many other cancers.

## Supplementary information

Supplementary Information

Supplementary Table 2

Supplementary Table 4
