# Supplementary material for: Genomic and transcriptomic profiling reveal molecular characteristics of parathyroid carcinoma
Source: Exp Mol Med. 2023 May 1;55(5):886–97. doi: 10.1038/s12276-023-00968-4 (PMC10238422; doi:10.1038/s12276-023-00968-4)

Supplementary Information

Genomic and transcriptomic profiling reveal molecular characteristics of parathyroid carcinoma

Se-Young Jo[1,†] , Namki Hong[2,†], Seunghyun Lee[2,3], Jong Ju Jeong[4], Jeongsoo Won[1], Jiho Park[1], Kijung Kim[5], Sang Kyum Kim[5], Sangwoo Kim[1,*], Yumie Rhee[2,*]

[1]Department of Biomedical Systems Informatics and Brain Korea 21 PLUS Project for Medical Science, Yonsei University College of Medicine, Seoul, Korea
[2]Department of Internal Medicine, Severance Hospital, Endocrine Research Institute, Yonsei University College of Medicine, Seoul, Korea
[3]Department of Internal Medicine, Wonju Severance Christian Hospital, Yonsei University Wonju College of Medicine, South Korea
[4]Department of Surgery, Severance Hospital, Yonsei University College of Medicine, Seoul, Korea
[5]Department of Pathology, Yonsei University College of Medicine, Seoul, Korea

†These authors contributed equally to this work.
* Corresponding authors

Contents

SUPPLEMENTARY TABLES **3**

SUPPLEMENTARY TABLE 1. SAMPLE INFORMATION 3

SUPPLEMENTARY TABLE 3. GSEA RESULTS (CARCINOMA AND ADENOMA) 5

SUPPLEMENTARY TABLE 5. GSEA RESULTS (*CDC73*^{MUT} AND *CDC73*^{WT} CARCINOMA) 6

SUPPLEMENTARY TABLE 6. CLINICAL CHARACTERISTICS OF NON-CARCINOMA GROUPS IN FIG. 5 7

SUPPLEMENTARY FIGURES **8**

SUPPLEMENTARY FIG. 1. AGE DISTRIBUTION AT SAMPLE ACQUISITION 8

SUPPLEMENTARY FIG. 2. MUTATIONAL SIGNATURES OF CARCINOMA SAMPLES BY *CDC73* MUTATION STATUS 9

SUPPLEMENTARY FIG. 3. PRE-PROCESSING OF RNA-SEQ DATA 10

SUPPLEMENTARY FIG. 4. FPKM COMPARISONS 11

SUPPLEMENTARY FIG. 5. NETWORK PLOT OF GSEA RESULT 12

SUPPLEMENTARY FIG. 6. DIFFERENTIALLY EXPRESSED GENES IN ADENOMA 13

SUPPLEMENTARY FIG. 7. GROUP-WISE COMPARISON OF *CDC73* EXPRESSION 14

SUPPLEMENTARY FIG. 8. WHOLE-CHROMOSOME B-ALLELE FREQUENCIES OF 10 CARCINOMAS 15

SUPPLEMENTARY FIG. 9. ALLELE-SPECIFIC COPY NUMBER STATUS OF 10 CARCINOMAS 16

SUPPLEMENTARY FIG. 10. ALLELIC IMBALANCE OF *CDC73* 17

SUPPLEMENTARY FIG. 11. CARCINOMA- AND ADENOMA-SPECIFIC DEG SELECTION AND CLUSTERING RESULTS WITH VARIOUS CUT-OFFS 18

SUPPLEMENTARY FIG. 12. *CDC73*^{MUT}- AND *CDC73*^{WT}-SPECIFIC DEG SELECTION AND CLUSTERING RESULTS WITH VARIOUS CUT-OFFS 19

SUPPLEMENTARY FIG. 13. DIFFERENTIALLY EXPRESSED GENES OF ADENOMA GROUP 1 AND GROUP 2 IN FIG. 5 20

SUPPLEMENTARY FIG. 14. WT1 IHC STAINING PICTURES 21

SUPPLEMENTARY FIG. 15. EXON USAGE OF *WT1* DETECTED BY DEXSEQ. 22

SUPPLEMENTARY FIG. 16. RARE GERMLINE VARIANTS PREDOMINANTLY FOUND IN CARCINOMA SAMPLES 23

SUPPLEMENTARY FIG. 17. IMMUNE CELL PROFILING RESULT PERFORMED WITH QUANTISEQ 24

Supplementary Tables

Supplementary Table 1. Sample Information

Information of samples used for this study. *CDC73* status for all samples are cross validated with not only the sequencing data indicated here, but also targeted sequencing and Sanger sequencing from the pathology department. *Note that RNA sequencing performed with two different library type, and number indicates the batches for the sequencing.

Sample Name	Sex	Age	Tumor Type	Sample acquisition		*CDC73* Status			
				DNA	RNA*	Germline mutation	Somatic mutation	LOH	Classification
P1_adenoma	Female	54	Adenoma	.	Total RNA (1)	None	None	N/A	.
P2_adenoma	Female	44	Adenoma	.	Total RNA (1)	None	None	N/A	.
P3_adenoma	Female	46	Adenoma	.	Total RNA (1)	None	None	N/A	.
P4_adenoma	Female	60	Adenoma	.	Total RNA (1)	None	None	N/A	.
P5_carcinoma	Female	45	Carcinoma	WES	Total RNA (1)	c.389_390del	c.87delG (frameshift)	No	$CDC73^{Mut}$
P6_carcinoma	Male	42	Carcinoma	WES	Total RNA (1)	c.687_688delAG (frameshift)	c.40delC (frameshift)	No	$CDC73^{Mut}$
P7_adenoma	Male	56	Adenoma	.	Total RNA (1)	None	None	N/A	.
P8_adenoma	Female	57	Adenoma	.	Total RNA (1)	None	None	N/A	.
P9_adenoma	Female	67	Adenoma	.	Total RNA (1)	None	None	N/A	.
P10_adenoma	Female	62	Adenoma	.	Total RNA (1)	None	None	N/A	.
P11_carcinoma	Female	41	Carcinoma	WES	Total RNA (1)	c.376C>T (stop gained)	c.128G>A (stop gained, COSV66465775)	No	$CDC73^{Mut}$
P22_carcinoma	Female	24	Carcinoma	WES	RNA Direct (2)	c.458T>A (stop gained)	c.75delG (frameshift)	Yes	$CDC73^{Mut}$
P23_adenoma	Female	35	Adenoma	.	RNA Direct (2)	None	None	N/A	.
P24_adenoma	Female	33	Adenoma	.	RNA Direct (2)	None	None	N/A	.
P25_adenoma	Male	80	Adenoma	.	RNA Direct (2)	None	None	N/A	.
P26_adenoma	Female	69	Adenoma	.	RNA Direct (2)	None	None	N/A	.
P27_carcinoma	Male	66	Carcinoma	.	RNA Direct (2)	None	c.162C>G (stop gained, COSV66466140)	Yes	$CDC73^{Mut}$
P28_adenoma	Female	59	Adenoma	.	RNA Direct (2)	None	None	N/A	.
P30_adenoma	Female	73	Adenoma	.	RNA Direct (2)	None	None	N/A	.
P31_adenoma	Female	75	Adenoma	.	RNA Direct (2)	None	None	N/A	.
P32_carcinoma	Female	25	Carcinoma	WES	RNA Direct (2)	None	c.155_168delGAGAGTACTACACA (frameshift)	Yes	$CDC73^{Mut}$
P37_adenoma	Male	64	Adenoma	.	RNA Direct (3)	None	None	N/A	.
P38_adenoma	Female	60	Adenoma	.	RNA Direct (3)	None	None	N/A	.

Sample Name	Sex	Age	Tumor Type	Sample acquisition		*CDC73* Status			
				DNA	RNA*	Germline mutation	Somatic mutation	LOH	Classification
P40_adenoma	Female	69	Adenoma	.	RNA Direct (3)	None	None	N/A	.
P41_adenoma	Female	64	Adenoma	.	RNA Direct (3)	None	None	N/A	.
P42_adenoma	Female	71	Adenoma	.	RNA Direct (3)	None	None	N/A	.
P45_adenoma	Female	71	Adenoma	.	RNA Direct (3)	None	None	N/A	.
P46_adenoma	Female	65	Adenoma	.	RNA Direct (3)	None	None	N/A	.
P47_adenoma	Female	75	Adenoma	.	RNA Direct (3)	None	None	N/A	.
P56_normal	Female	45	Normal	.	RNA Direct (4)	None	None	N/A	.
P57_normal	Female	27	Normal	.	RNA Direct (4)	None	None	N/A	.
P58_normal	Female	30	Normal	.	RNA Direct (4)	None	None	N/A	.
P59_normal	Female	31	Normal	.	RNA Direct (4)	None	None	N/A	.
P60_normal	Female	32	Normal	.	RNA Direct (4)	None	None	N/A	.
P62_normal	Female	32	Normal	.	RNA Direct (4)	None	None	N/A	.
P63_normal	Female	56	Normal	.	RNA Direct (4)	None	None	N/A	.
P65_carcinoma	Female	24	Carcinoma	WES	RNA Direct (4)	None	None	No	$CDC73^{WT}$
P66_carcinoma	Female	57	Carcinoma	WES	RNA Direct (4)	None	None	No	$CDC73^{WT}$
P67_adenoma	Female	63	Adenoma	.	RNA Direct (5)	None	None	N/A	.
P68_carcinoma	Female	59	Carcinoma	WES	RNA Direct (5)	None	None	Yes	$CDC73^{WT}$
P69_normal	Female	40	Normal	.	RNA Direct (5)	None	None	N/A	.
P70_adenoma	Female	56	Adenoma	.	RNA Direct (5)	None	None	N/A	.
P71_normal	Male	54	Normal	.	RNA Direct (5)	None	None	N/A	.
P72_adenoma	Female	65	Adenoma	.	RNA Direct (5)	None	None	N/A	.
P73_normal	Male	37	Normal	.	RNA Direct (5)	None	None	N/A	.
P74_adenoma	Female	67	Adenoma	.	RNA Direct (5)	None	None	N/A	.
P75_carcinoma	Male	16	Carcinoma	WES	RNA Direct (5)	c.389_390del	None	No	$CDC73^{Mut}$
P76_adenoma	Female	56	Adenoma	.	RNA Direct (5)	None	None	N/A	.
P77_carcinoma	Male	64	Carcinoma	WES	.	None	None	Yes	$CDC73^{WT}$
P79_carcinoma	Female	43	Carcinoma	.	RNA Direct (6)	None	None	N/A	$CDC73^{WT}$

Supplementary Table 3. GSEA results (carcinoma and adenoma)

Significantly enriched gene sets at nominal p value <1% and FDR <25%

Carcinoma to normal

Name	Normalized Enrichment Score	Nominal p-value	FDR
HALLMARK_E2F_TARGETS	2.389	<0.001	<0.001
HALLMARK_G2M_CHECKPOINT	2.380	<0.001	<0.001
HALLMARK_MYC_TARGETS_V2	2.240	<0.001	<0.001
HALLMARK_MITOTIC_SPINDLE	2.158	<0.001	<0.001
HALLMARK_MYC_TARGETS_V1	2.120	<0.001	<0.001
HALLMARK_HEDGEHOG_SIGNALING	1.912	<0.001	<0.001
HALLMARK_MTORC1_SIGNALING	1.902	<0.001	<0.001
HALLMARK_EPITHELIAL_MESENCHYMAL_TRANSITION	1.784	<0.001	<0.001
HALLMARK_GLYCOLYSIS	1.582	<0.001	0.015
HALLMARK_COAGULATION	1.571	<0.001	0.015
HALLMARK_UNFOLDED_PROTEIN_RESPONSE	1.527	0.005	0.023
HALLMARK_APICAL_JUNCTION	1.509	0.003	0.027
HALLMARK_DNA_REPAIR	1.457	0.008	0.034
HALLMARK_KRAS_SIGNALING_UP	1.420	0.008	0.042

Adenoma to normal

Name	Normalized Enrichment Score	Nominal p-value	FDR
HALLMARK_MITOTIC_SPINDLE	1.989	<0.001	0.001
HALLMARK_MYC_TARGETS_V2	1.845	0.003	0.003
HALLMARK_HEDGEHOG_SIGNALING	1.807	0.003	0.003
HALLMARK_E2F_TARGETS	1.642	<0.001	0.010
HALLMARK_G2M_CHECKPOINT	1.577	<0.001	0.016
HALLMARK_MYC_TARGETS_V1	1.419	0.004	0.050
HALLMARK_MYOGENESIS	-1.490	<0.001	0.054
HALLMARK_HYPOXIA	-1.499	0.003	0.057
HALLMARK_ADIPOGENESIS	-1.511	<0.001	0.061
HALLMARK_INFLAMMATORY_RESPONSE	-1.516	0.005	0.070
HALLMARK_KRAS_SIGNALING_DN	-1.520	0.003	0.089
HALLMARK_ALLOGRAFT_REJECTION	-1.793	<0.001	0.005
HALLMARK_TNFA_SIGNALING_VIA_NFKB	-2.182	<0.001	<0.001

Supplementary Table 5. GSEA results (*CDC73*^{Mut} and *CDC73*^{WT} carcinoma)

Significantly enriched gene sets at nominal p value <1% and FDR <25%

***CDC73*^{Mut} carcinoma to normal**

Name	Normalized Enrichment Score	Nominal p-value	FDR
HALLMARK_E2F_TARGETS	2.508	<0.001	<0.001
HALLMARK_G2M_CHECKPOINT	2.453	<0.001	<0.001
HALLMARK_MYC_TARGETS_V1	2.396	<0.001	<0.001
HALLMARK_MYC_TARGETS_V2	2.332	<0.001	<0.001
HALLMARK_MITOTIC_SPINDLE	2.239	<0.001	<0.001
HALLMARK_MTORC1_SIGNALING	1.842	<0.001	<0.001
HALLMARK_HEDGEHOG_SIGNALING	1.748	<0.001	0.002
HALLMARK_COAGULATION	1.562	<0.001	0.019
HALLMARK_EPITHELIAL_MESENCHYMAL_TRANSITION	1.381	0.01	0.083
HALLMARK_XENOBIOTIC_METABOLISM	1.347	0.01	0.08

***CDC73*^{WT} carcinoma to normal**

Name	Normalized Enrichment Score	Nominal p-value	FDR
HALLMARK_EPITHELIAL_MESENCHYMAL_TRANSITION	2.229	<0.001	<0.001
HALLMARK_OXIDATIVE_PHOSPHORYLATION	2.036	<0.001	<0.001
HALLMARK_MYC_TARGETS_V2	1.932	<0.001	<0.001
HALLMARK_MTORC1_SIGNALING	1.879	<0.001	0.001
HALLMARK_HEDGEHOG_SIGNALING	1.839	0.002	0.002
HALLMARK_E2F_TARGETS	1.826	<0.001	0.002
HALLMARK_G2M_CHECKPOINT	1.825	<0.001	0.001
HALLMARK_ALLOGRAFT_REJECTION	1.731	<0.001	0.004
HALLMARK_UNFOLDED_PROTEIN_RESPONSE	1.699	<0.001	0.005
HALLMARK_KRAS_SIGNALING_UP	1.666	<0.001	0.006
HALLMARK_GLYCOLYSIS	1.620	<0.001	0.009
HALLMARK_REACTIVE_OXYGEN_SPECIES_PATHWAY	1.608	0.005	0.009
HALLMARK_APICAL_JUNCTION	1.593	0.000	0.010
HALLMARK_IL6_JAK_STAT3_SIGNALING	1.591	0.000	0.009
HALLMARK_MYOGENESIS	1.569	0.002	0.011
HALLMARK_APOPTOSIS	1.549	0.005	0.013
HALLMARK_COAGULATION	1.529	0.002	0.015

Supplementary Table 6. Clinical characteristics of non-carcinoma groups in Fig. 5

Non-carcinoma group 1 refers to the left branch on the non-carcinoma cluster in Fig. 5, while the group 2 refers to the right one. Note that there was no significant difference found between adenomas in group 1 and group 2 in any category.

	**Non-carcinoma group 1**			**Non-carcinoma group 2**	**P value** (group 1 vs. 2)
	Total (n=16)	Normal in group 1 (n=10)	Adenoma in group 1 (n=6)	Total (n=22)	
Age	50 [32-62]	35 [31-45]	68 [59-75]*	63 [56-67]*	0.047
Women, n(%)	12 (75)	8 (80)	4 (67)	21 (95)	0.066
Preoperative PTH, pg/mL	48 [33-95]	35 [26-45]	105 [93-127]*	119 [88-217]*	<0.001
Corrected calcium, mg/dL	10 [9.5-10.9]	9.7 [9.4-9.9]	11.5 [10.5-11.9]*	10.8 [10.4-11.3]*	0.005
Inorganic phosphorus, mg/dL	3.5 ± 0.6	3.9 ± 0.5	3.1 ± 0.7*	2.9 ± 0.5*	0.002
Tumor long diameter, cm		Not applicable	1.2 [0.8-1.7]	1.3 [0.9-2.1]	
Disease group					<0.001
Adenoma, n (%)	6 (37)	0 (0)	6 (100)	22 (100)	
Normal, n (%)	10 (63)	10 (100)	0 (0)	0 (0)	

*P<0.05 vs. normal in group 1

No statistically significant differences of clinicopathologic features were observed between adenomas in group 1 and group 2.

Supplementary Figures

Supplementary Fig. 1. Age distribution at sample acquisition

Age distribution was the significantly younger in carcinoma (median age = 42.5, p = 0.0009, Mann-Whitney test) than adenoma (median age = 63.5) groups. Normal group patients were the youngest (median age = 34.5) at the time of sample acquisition.

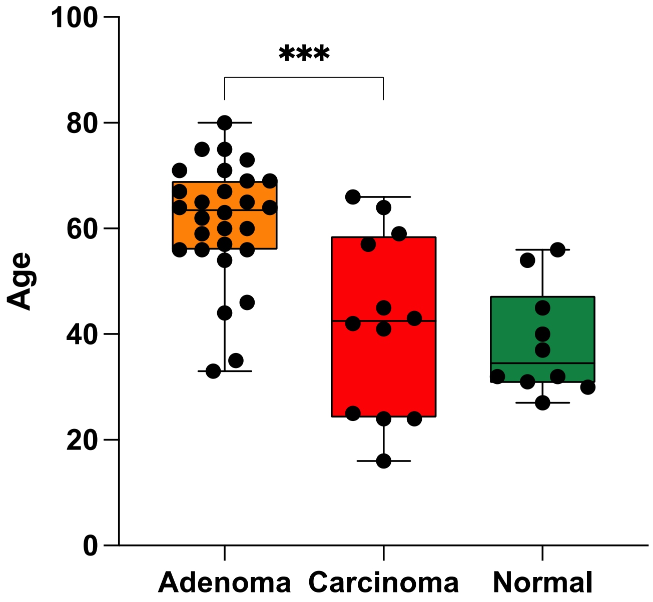

Supplementary Fig. 2. Mutational signatures of carcinoma samples by *CDC73* mutation status

(a) Mutational signatures of *CDC73^{Mut}* samples. (b) Estimated exposures on every *CDC73^{Mut}* samples. Note that SBS1 affected only on P5. (c) Mutational signature of *CDC73^{WT}* samples. Only one signature, SBS6, has been detected upon *CDC73^{WT}* samples. (d) Estimated exposures on every *CDC73^{WT}* samples.

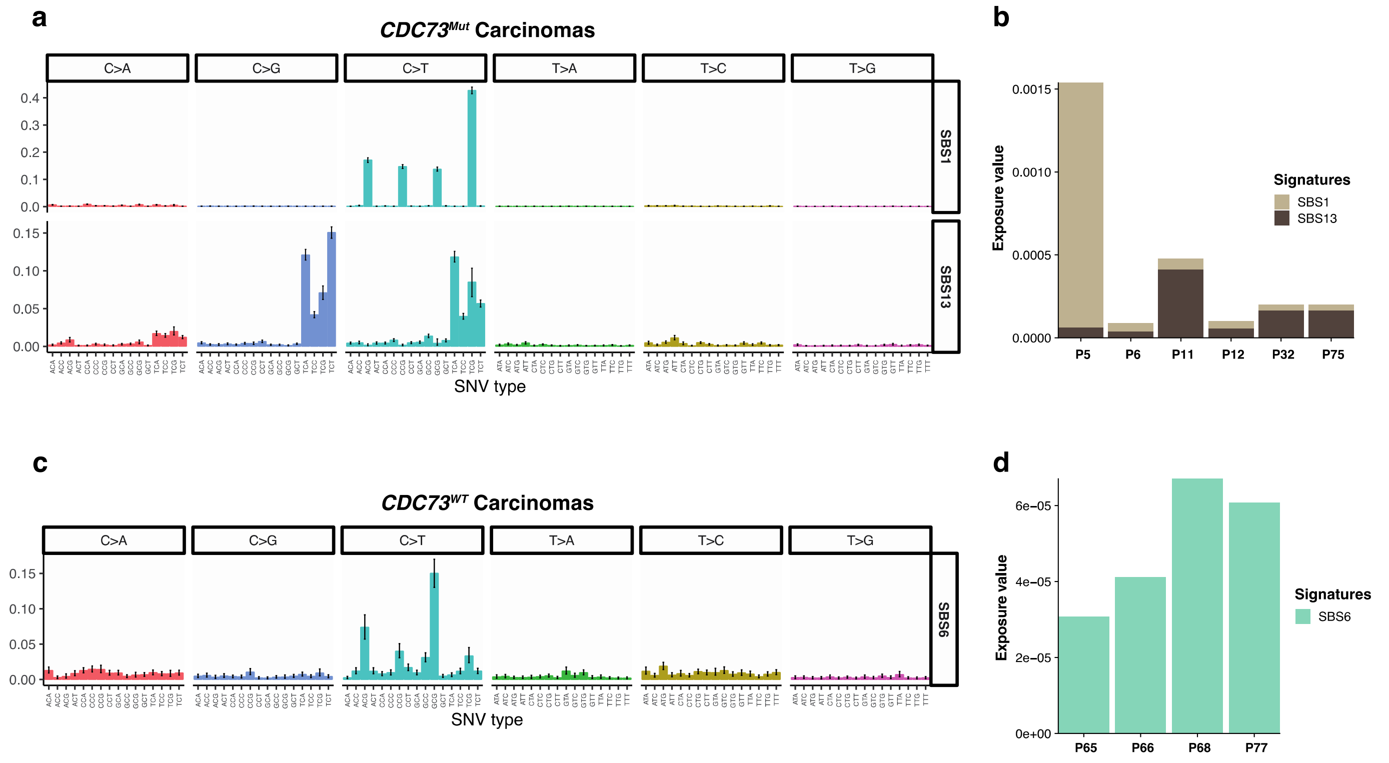

Supplementary Fig. 3. Pre-processing of RNA-seq data

(a) Schematic overview of transcriptomics analysis pipeline. (b) FPM distribution of all genes in three groups. A uniform distribution was successfully obtained through target gene restriction and normalization. (c) PCA plot before batch-effect removal. (d) PCA plot after batch-effect removal.

a

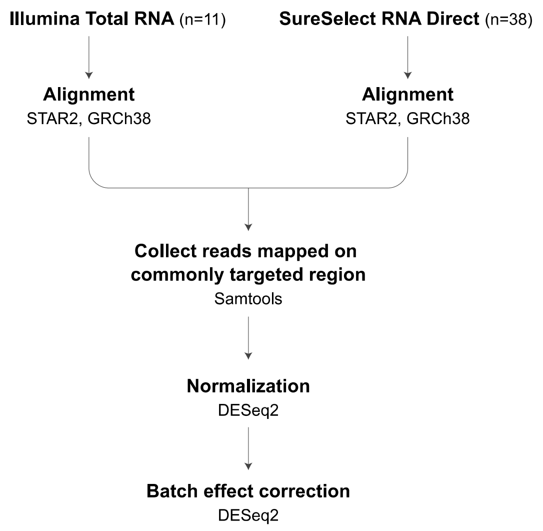

b

[redacted]

c

[redacted]

d

[redacted]

Supplementary Fig. 4. FPKM Comparisons

FPKM comparisons of all genes in three groups. The correlation between normal and adenoma was the highest ($r^2 = 0.982$) as expected, and that of normal and carcinoma was the lowest ($r^2 = 0.943$).

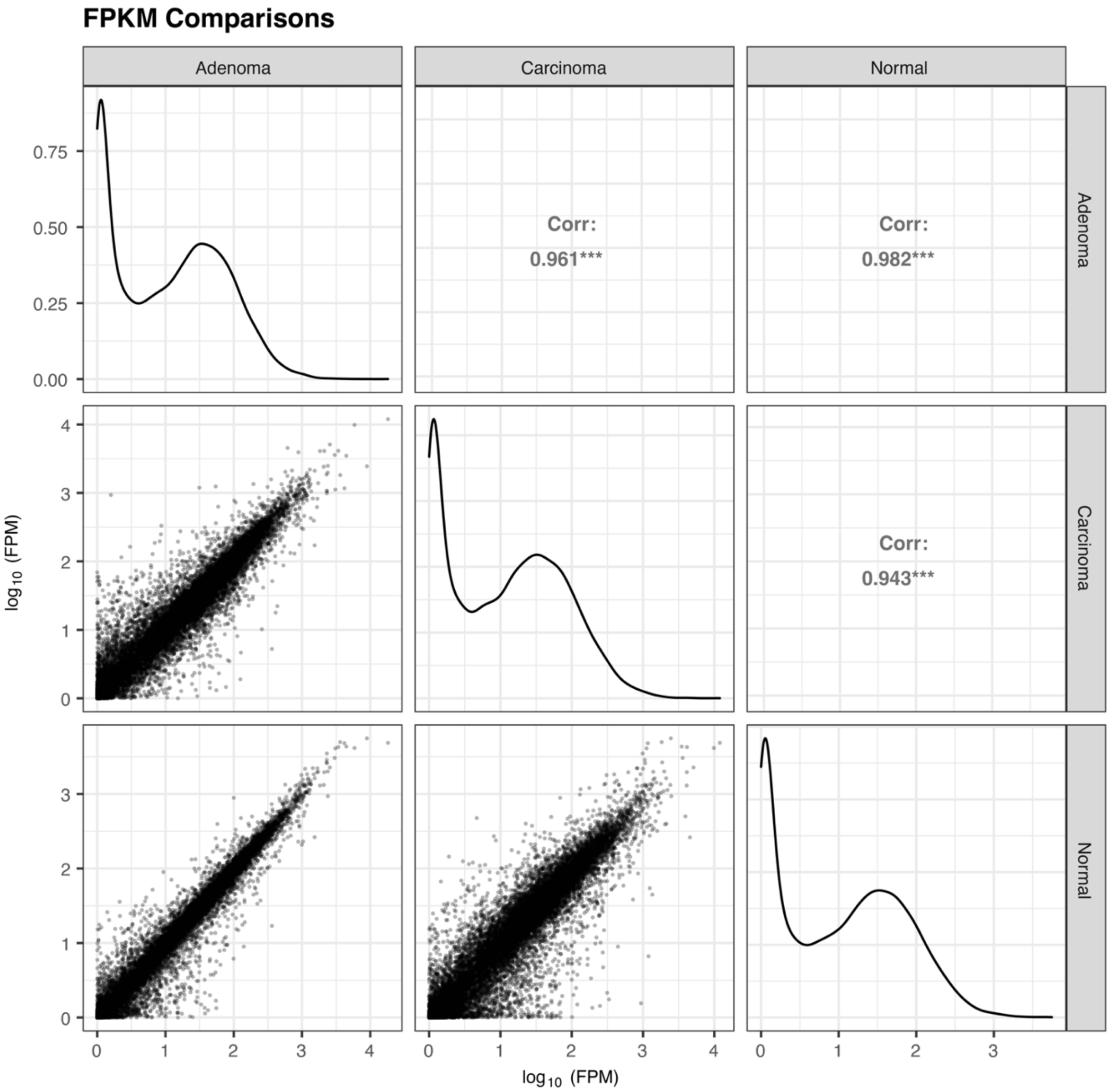

Supplementary Fig 5. Network plot of GSEA result

Significant GSEA results shown with the genes belonging to each term.

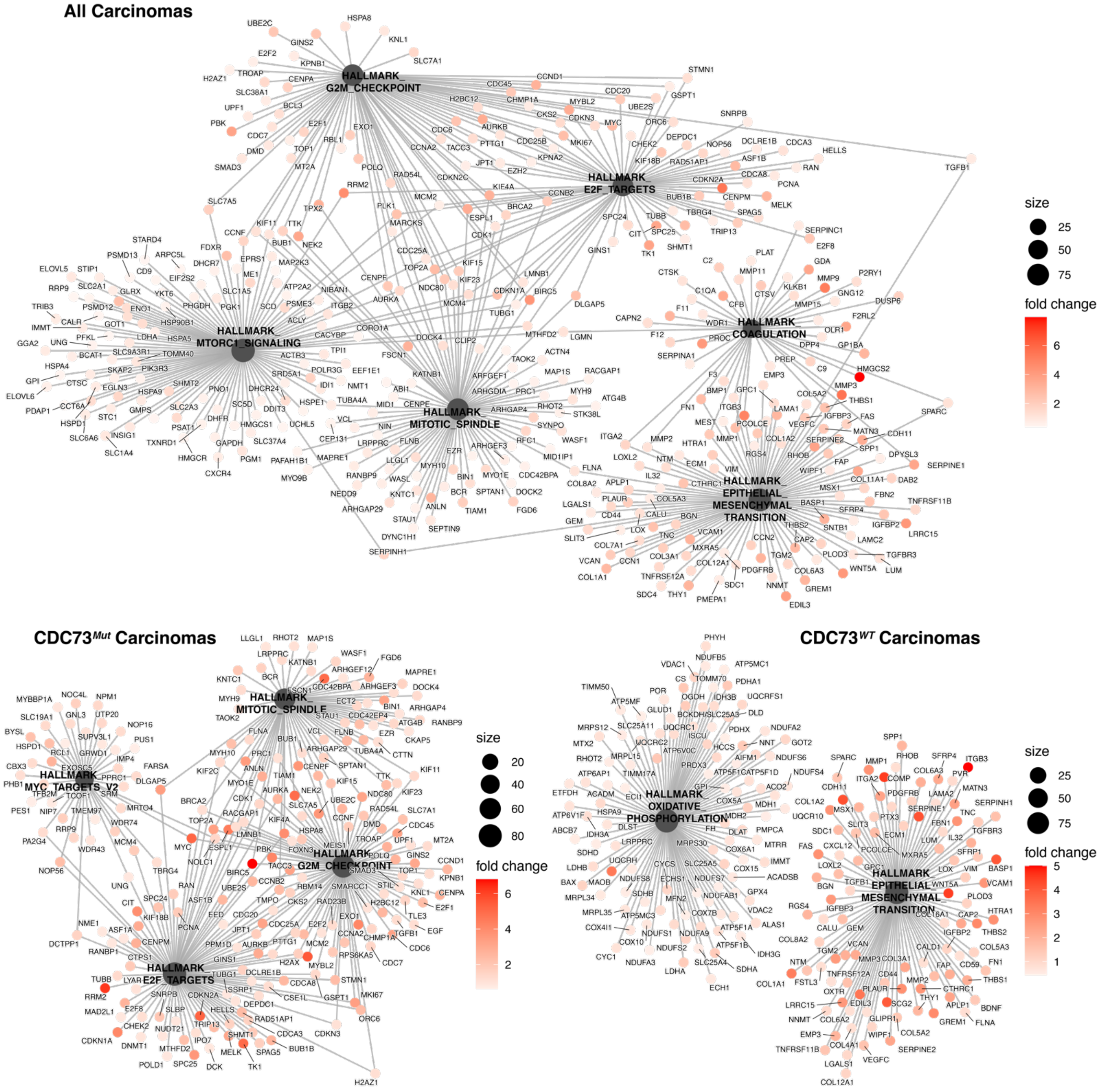

Supplementary Fig. 6. Differentially expressed genes in adenoma

(a) Differentially expressed genes of adenoma with a |fold-change| > 1.5 cutoff. (b) GO enrichment result of Up-DEGs of adenoma. (Red dots in Supplementary Fig. 6a) (c) Gene components belong to each term found in Supplementary Fig. 6b.

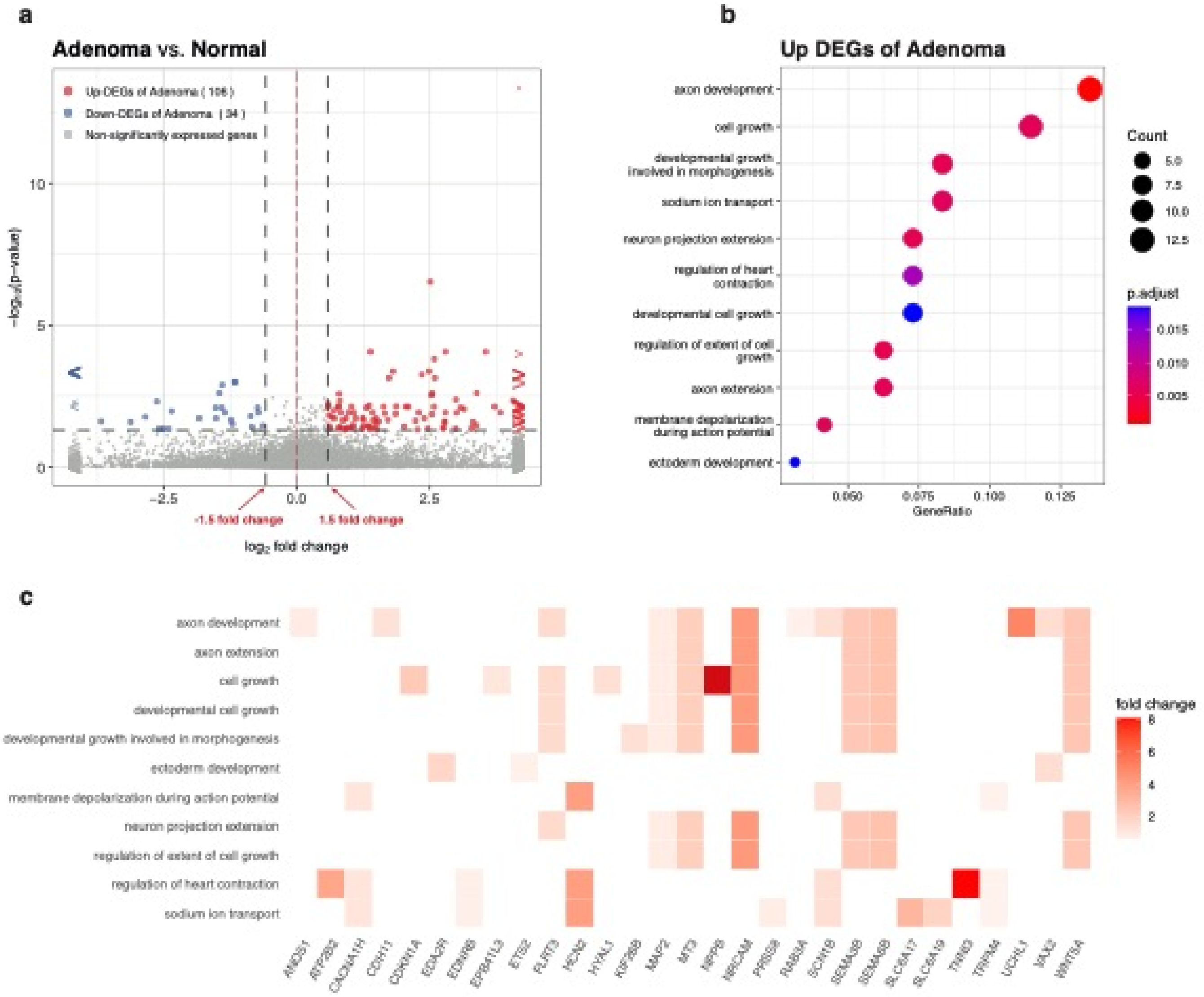

Supplementary Fig. 7. Group-wise comparison of *CDC73* expression

CDC73 was found not to be a significant DEG.

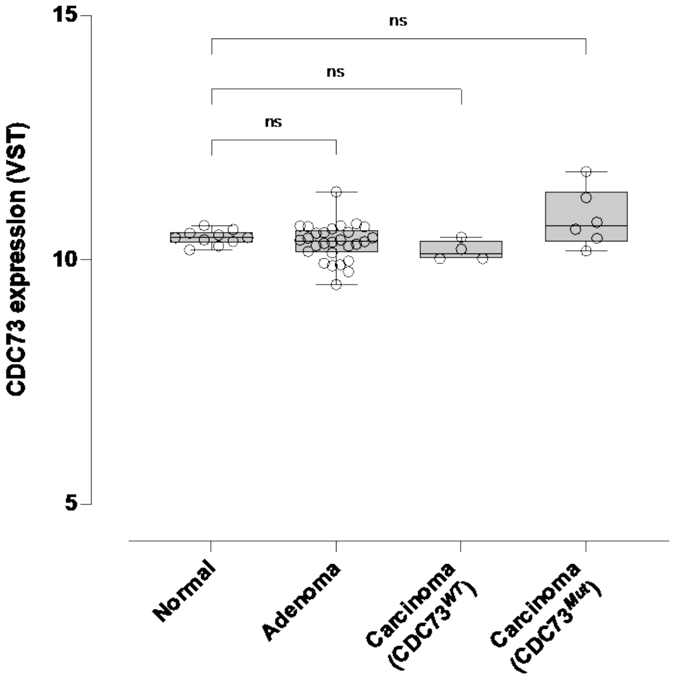

Supplementary Fig. 8. Whole-chromosome B-allele frequencies of 10 carcinomas

B-allele frequencies of all hetero-SNPs throughout the whole-chromosome observed by PureCN (v.2.1.2). The expected B-allele frequency of hetero-SNP is 0.5 in normal copy number condition (ploidy = 2), and if two values symmetrically diverging at 0.5 are observed, it can be assumed as evidence of asymmetric copy number variation. Red arrow indicates a genomic region that contains *CDC73* gene.

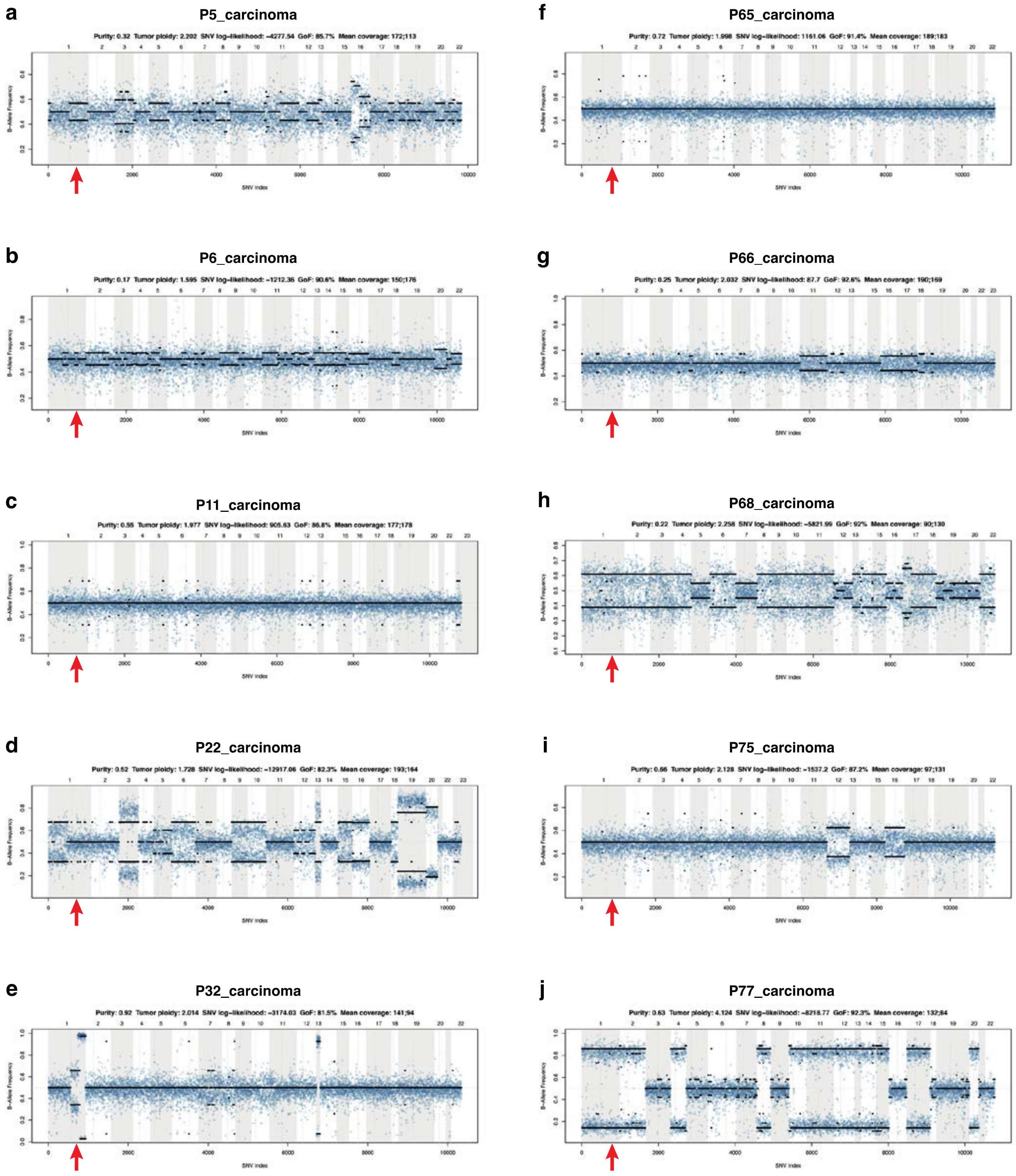

Supplementary Fig. 9. Allele-specific copy number status of 10 carcinomas

Allele-specific copy number calling result from Sequenza (v.3.0.0). Red line represents major copy number, and blue line represents minor copy number referred from the combination of B-allele frequency and relative read depth. Adding the value of the red and blue line means the total copy number of the given genomic position. Red arrow indicates a genomic region that contains *CDC73* gene.

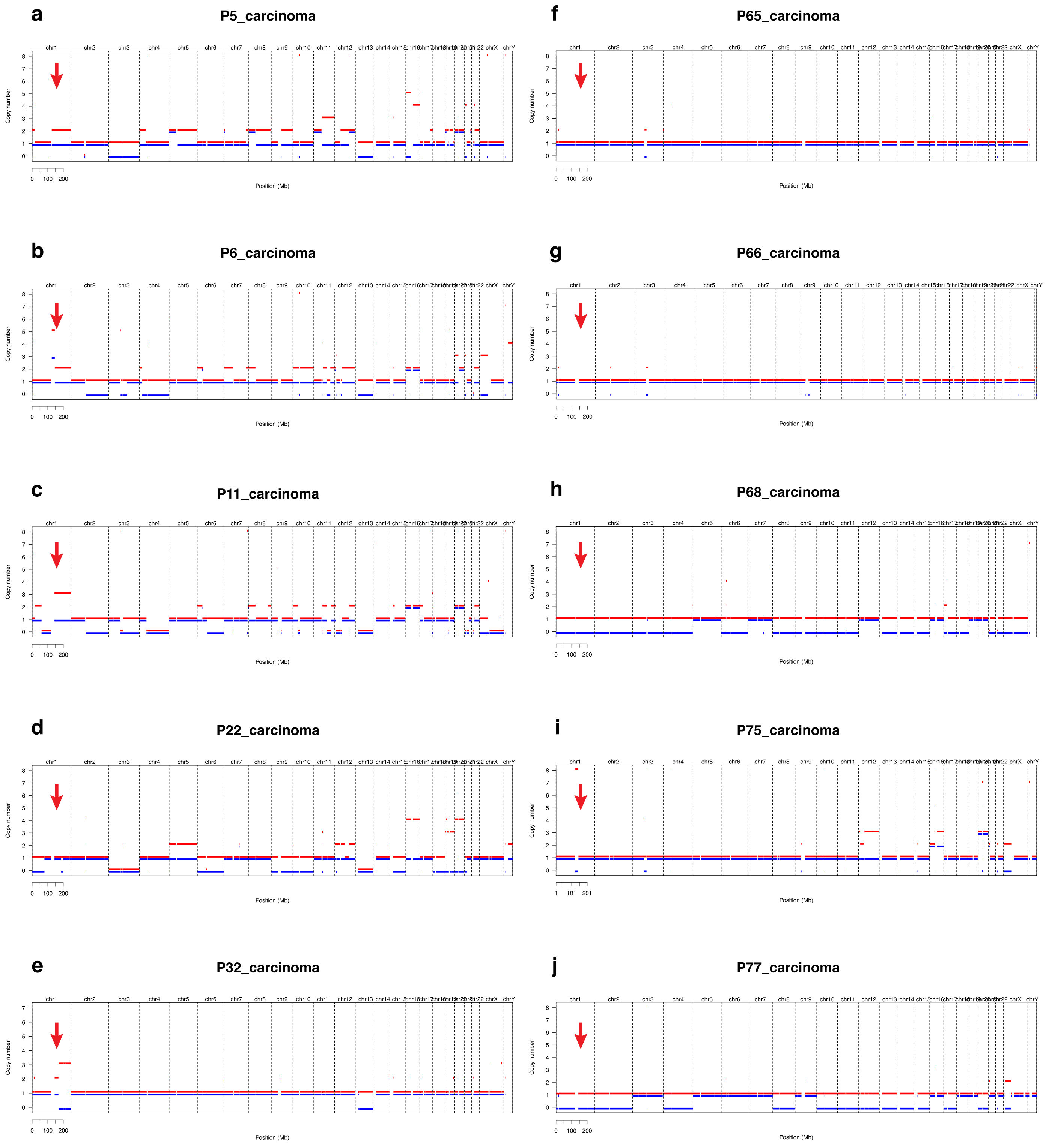

Supplementary Fig. 10. Allelic imbalance of *CDC73*

Uncorrected raw values of *CDC73* variant allele-frequencies (VAFs) traced through Normal DNA, Tumor DNA, and Tumor RNA. In the two-hit mutant group, the decrease in germline VAF as much as the increase in somatic VAF is strong evidence that the two variants exist in different copies. Furthermore, the expression of somatic mutated copies is commonly upregulated, and in the case of P75, the expression of germline mutated copy is suppressed even though there is no second-hit suggesting that the down-regulation is prior to the second-hit event or copy number aberration.

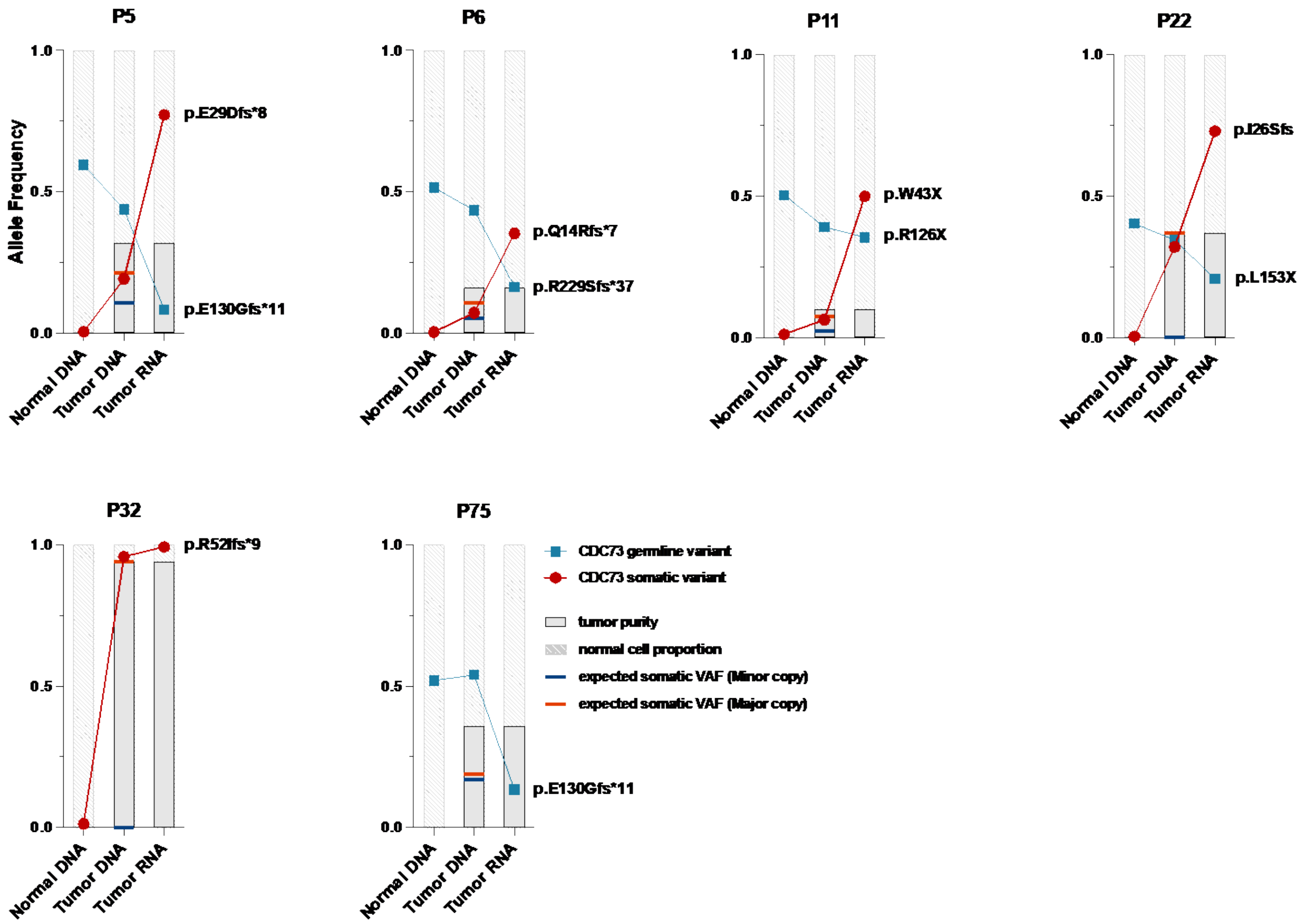

Supplementary Fig. 11. Carcinoma- and adenoma-specific DEG selection and clustering results with various cut-offs

DEG selection with (a, b) $p_{adj} < 0.05$ and $|\log_2 \text{fold change}| > 1$ criteria, (c, d) $p_{adj} < 0.05$ & $|\log_2 \text{fold change}| > 2$ criteria, (e, f) $p_{adj} < 0.01$ & $|\log_2 \text{fold change}| > 1$ criteria, (g, h) $p_{adj} < 0.01$ & $|\log_2 \text{fold change}| > 2$ criteria.

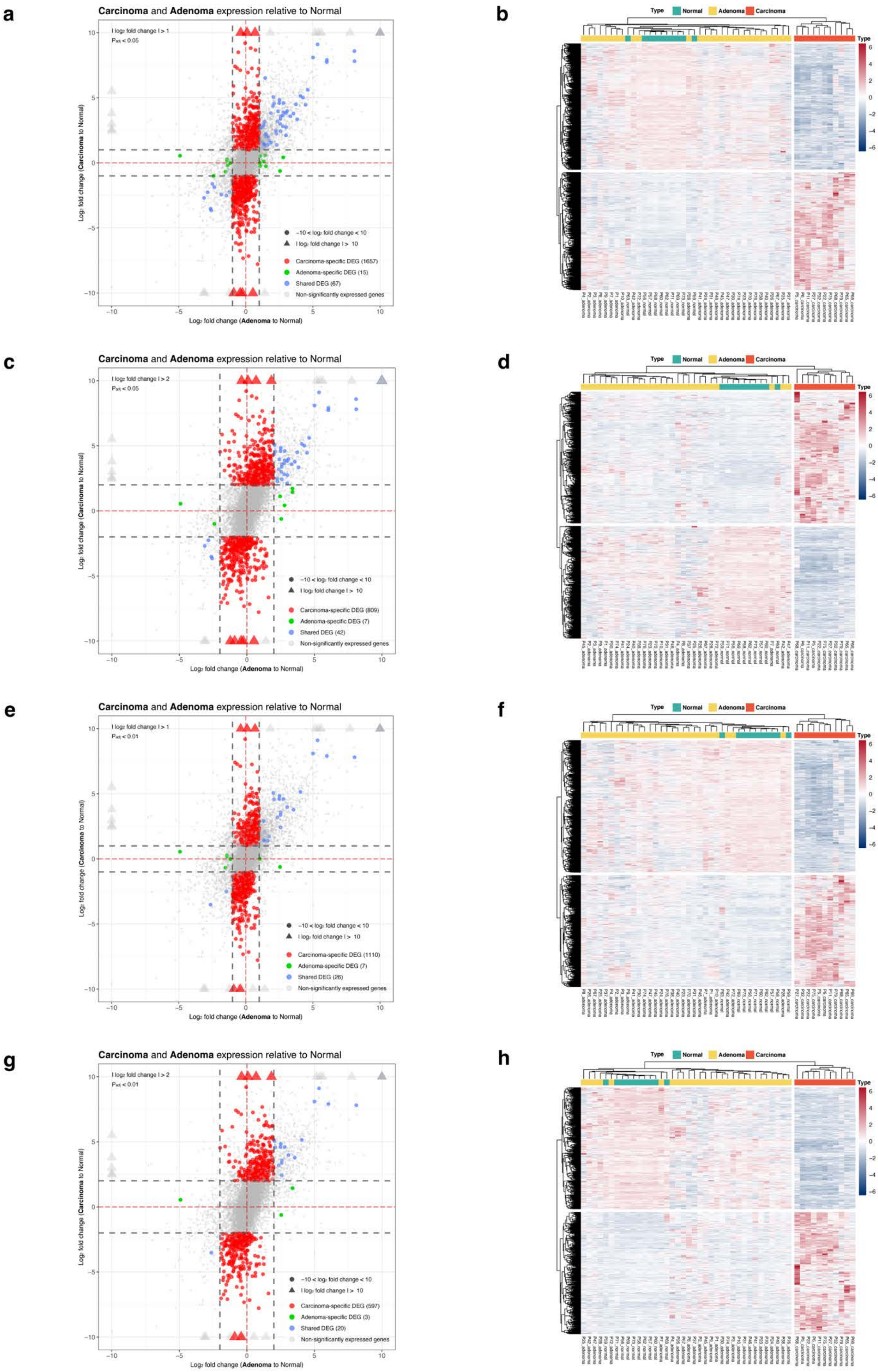

Supplementary Fig. 12. *CDC73^{Mut}*- and *CDC73^{WT}*-specific DEG selection and clustering results with various cut-offs

DEG selection with (a, b) $p_{adj} < 0.05$ and |$\log_2$ fold change| >1 criteria, (c, d) $p_{adj} < 0.05$ & |$\log_2$ fold change| >2 criteria, (e, f) $p_{adj} < 0.01$ & |$\log_2$ fold change| >1 criteria, (g, h) $p_{adj} < 0.01$ & |$\log_2$ fold change| >2 criteria.

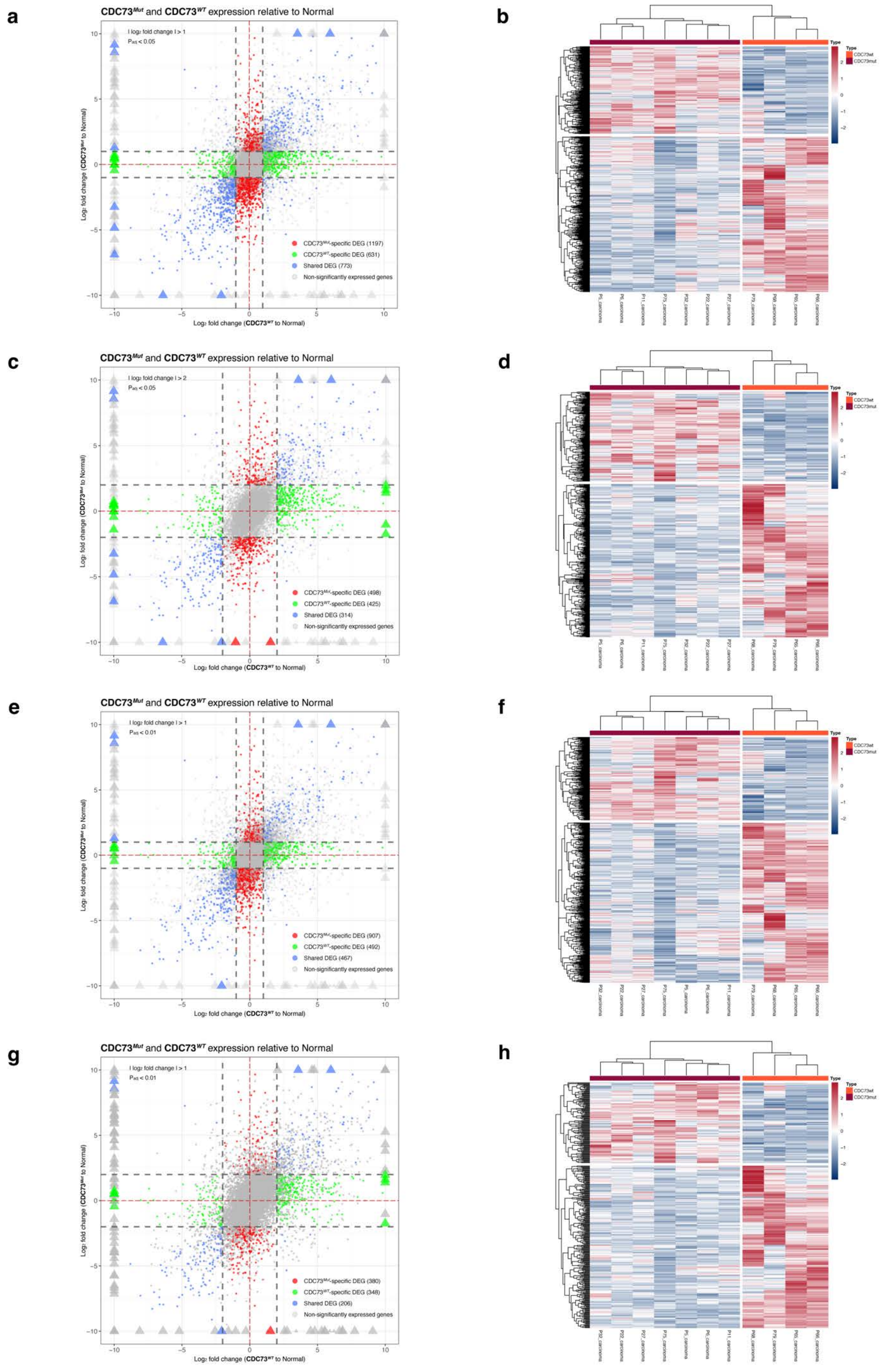

Supplementary Fig. 13. Differentially expressed genes of adenoma group 1 and group 2 in Fig. 5

DEGs of adenoma group 1 and group 2 at the criteria of $p_{adj} < 0.05$ and $|\log_2 \text{fold change}| > 1$.

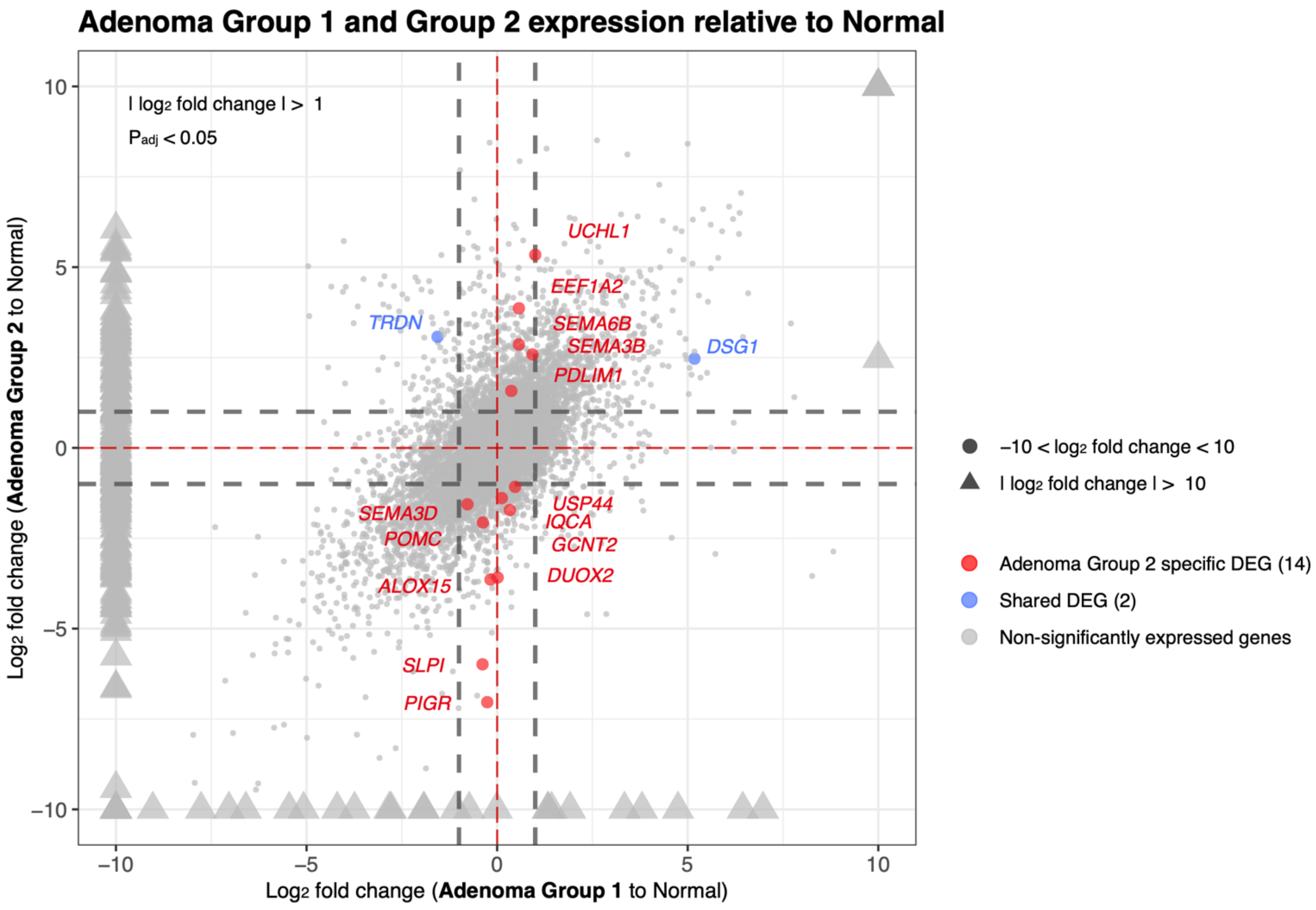

Supplementary Fig. 14. WT1 IHC staining pictures

5 out of 6 *CDC73*^{Mut} carcinoma (83.3%) have been stained with the WT1 antibody, and none of the adenoma or *CDC73*^{WT} carcinoma has been stained positively.

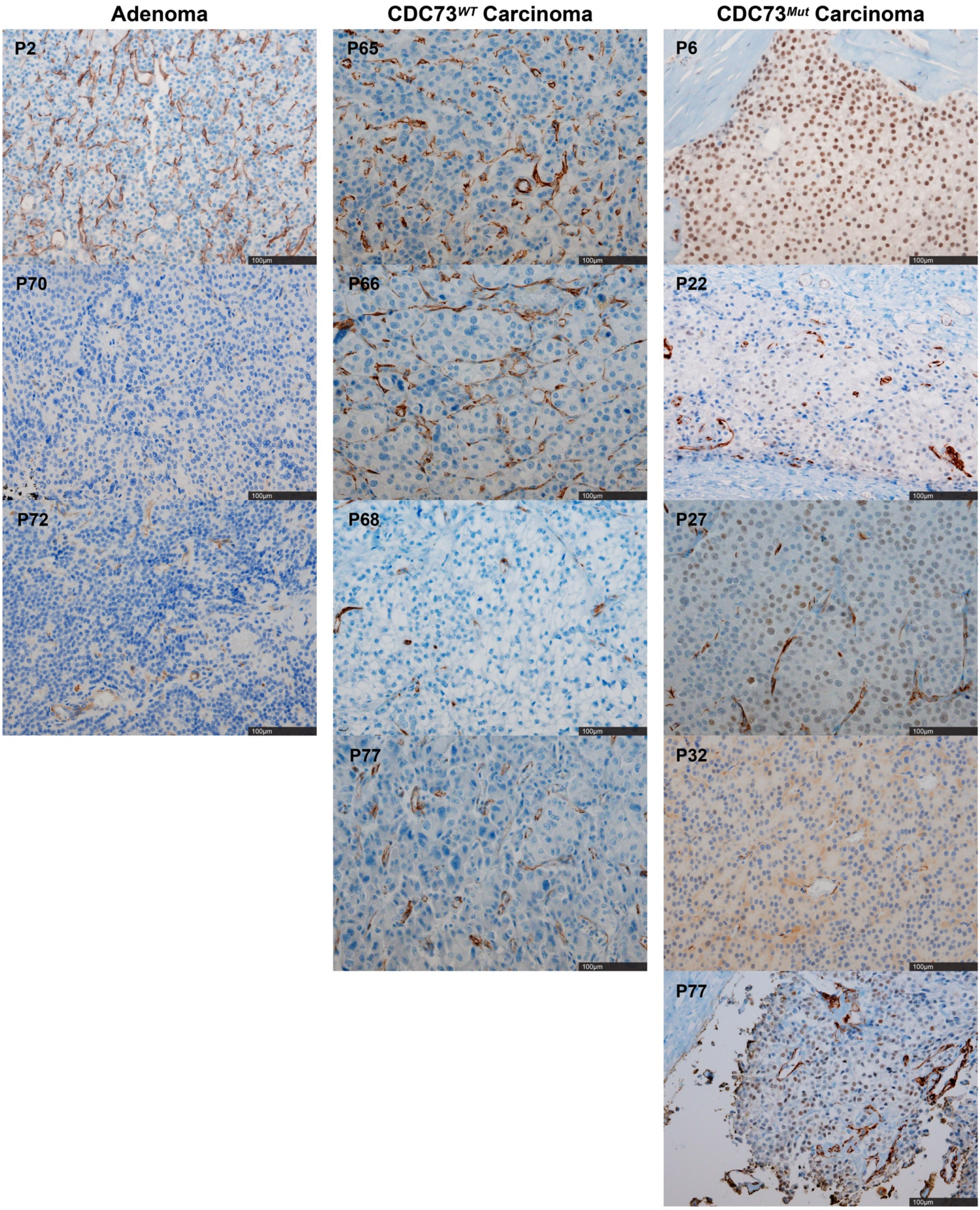

Supplementary Fig. 15. Exon usage of *WT1* detected by DEXSeq.

Each line indicates relative expression of corresponding exons. *CDC73*^{Mut} carcinomas revealed to use all of the exons of canonical *WT1* transcript, ESNT00000452863 (red lines). Exon 4 skipping was not detected in our cases of *CDC73*^{Mut} carcinoma. The exon usage of *CDC73*^{WT} carcinoma was not clearly detected because of its low coverage (blue lines), as well as adenoma and normal (not shown here).

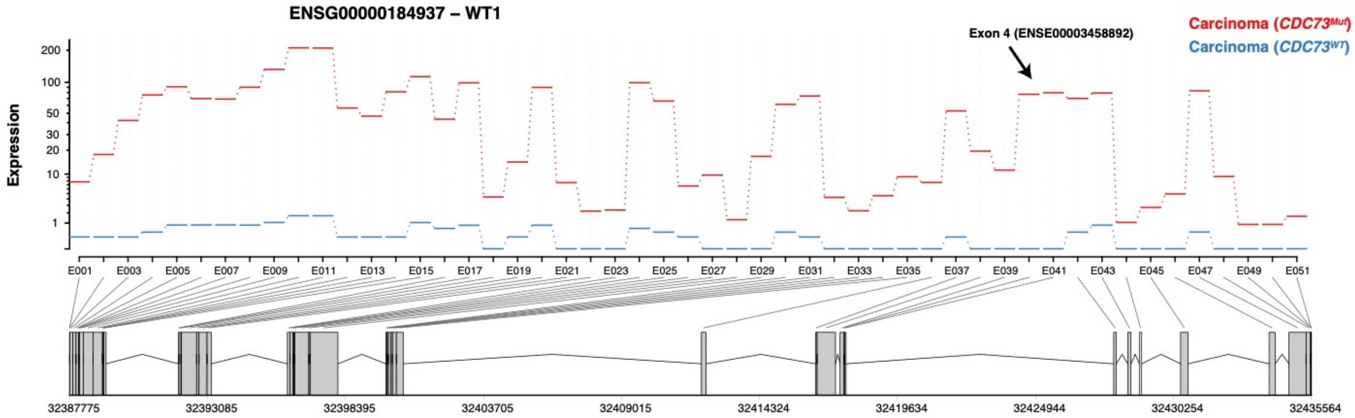

Supplementary Fig. 16. Rare germline variants predominantly found in carcinoma samples

Among the rare (MAF < 0.01) germline variants called from transcriptome sequencing data by Strelka2 RNA mode, mutated genes predominantly found in carcinoma group were selected. All variants plotted on the carcinoma group were manually cross checked in the WES dataset.

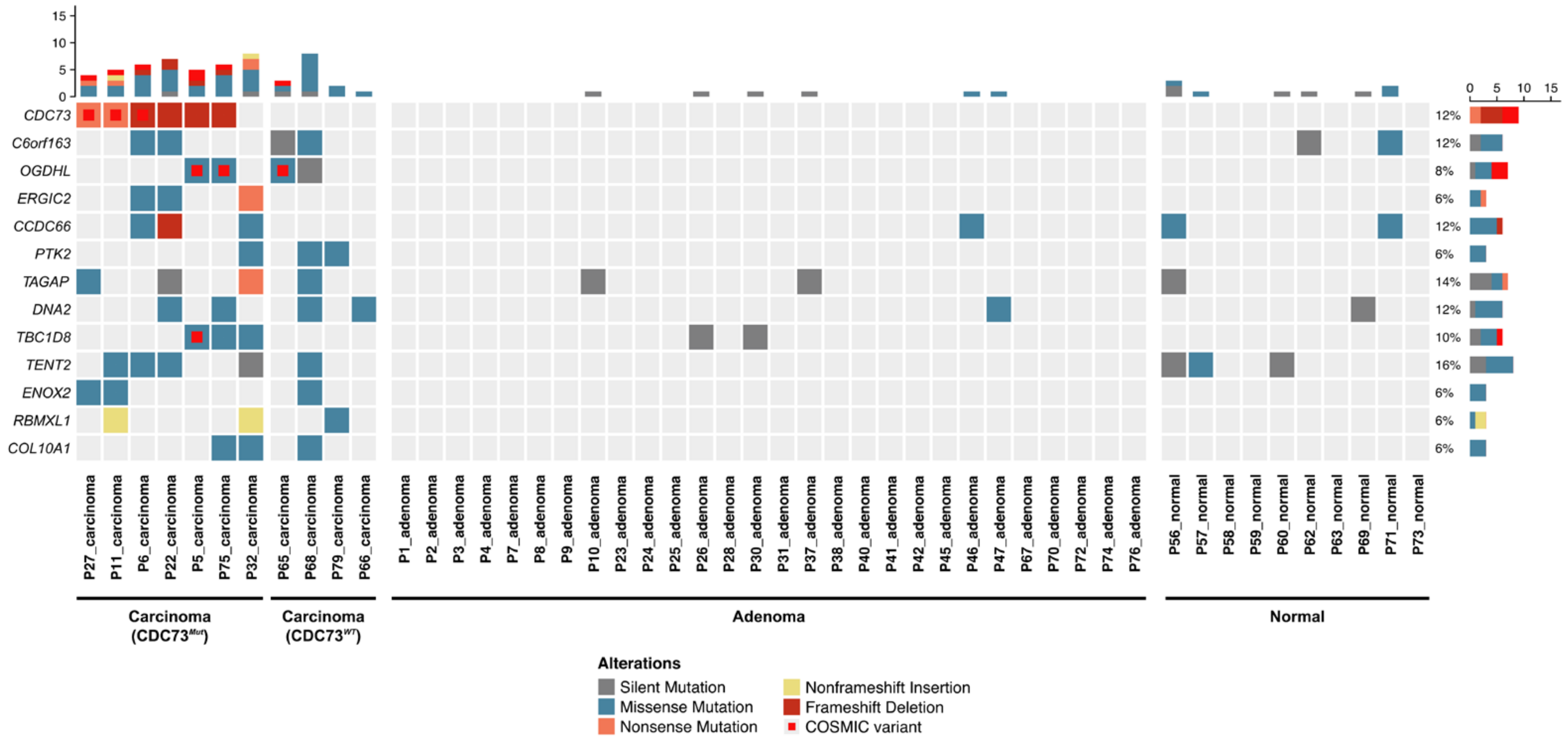

Supplementary Fig. 17. Immune cell profiling result performed with quanTIseq

Estimated immune cell fractions of adenoma and *CDC73*^{WT} carcinoma didn't showed significant difference to the normal cell, whereas *CDC73*^{Mut} carcinoma showed significantly low faction of immune cells (bottom right). By looking at the individual immune cell types, along with the NK cell and CD8+ T cell, T_{reg} was also observed to be significantly low in *CDC73*^{Mut} carcinoma.

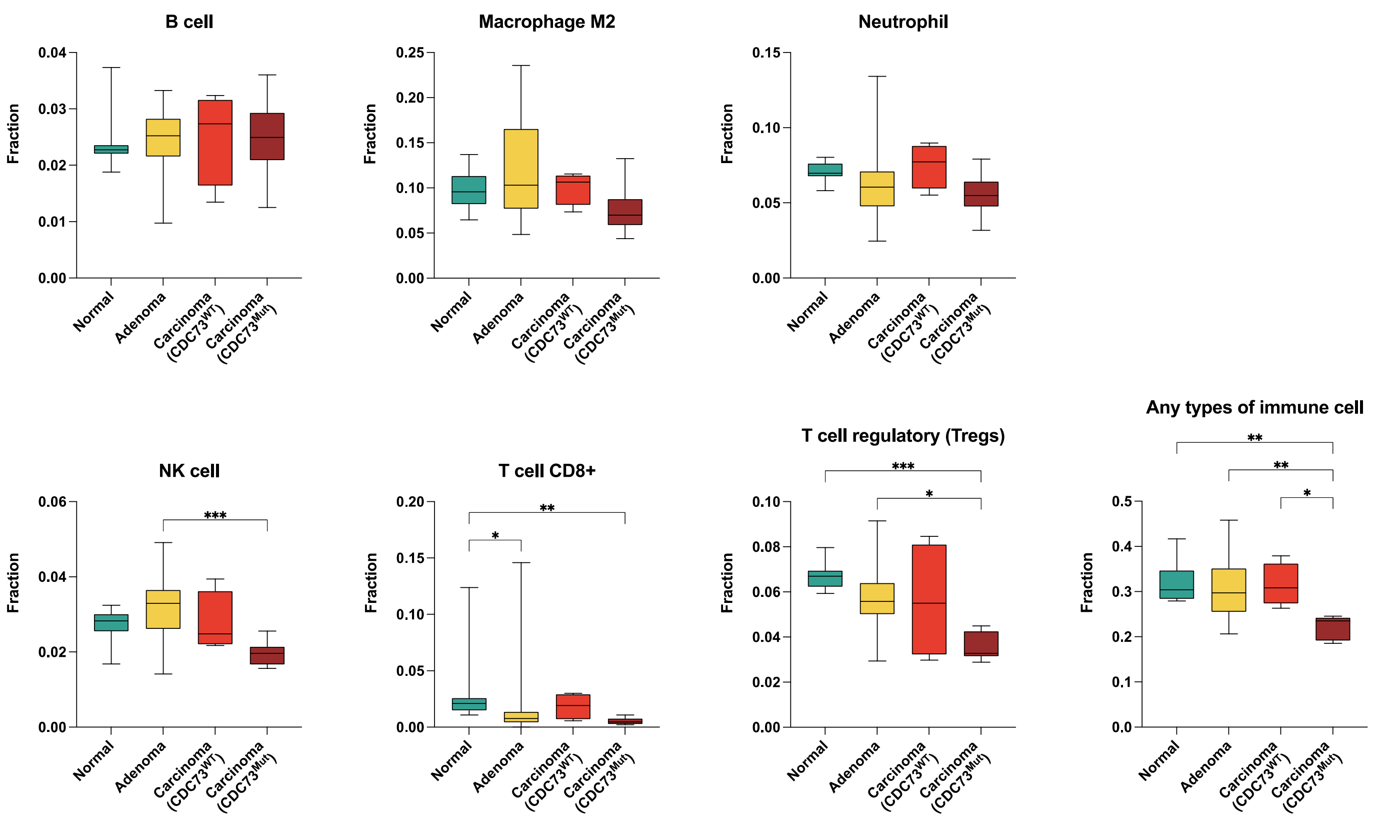

Supplement: Supplementary file 1 — Supplementary Information [file 12276_2023_968_MOESM1_ESM.pdf]
